# Accumulation of Per- and Polyfluoroalkyl Substances (PFAS) in Coastal Sharks from Contrasting Marine Environments: The New York Bight and The Bahamas

**DOI:** 10.1021/acs.est.4c02044

**Published:** 2024-07-12

**Authors:** Cheng-Shiuan Lee, Oliver N. Shipley, Xiayan Ye, Nicholas S. Fisher, Austin J. Gallagher, Michael G. Frisk, Brendan S. Talwar, Eric V.C. Schneider, Arjun K. Venkatesan

**Affiliations:** † Research Center for Environmental Changes, Academia Sinica, Taipei 115, Taiwan; ‡ School of Marine and Atmospheric Sciences, Stony Brook University, Stony Brook, New York 11794, United States; § New York State Center for Clean Water Technology, Stony Brook University, Stony Brook, New York 11794, United States; ∥ Beneath the Waves, Boston, Massachusetts 02129, United States; ⊥ Cape Eleuthera Institute, Rock Sound, Eleuthera 26029, The Bahamas; # Department of Civil and Environmental Engineering, New Jersey Institute of Technology, Newark, New Jersey 07102, United States

**Keywords:** elasmobranch, emerging contaminants, PFAS isomer, stable isotope, mercury, tolerable weekly intake

## Abstract

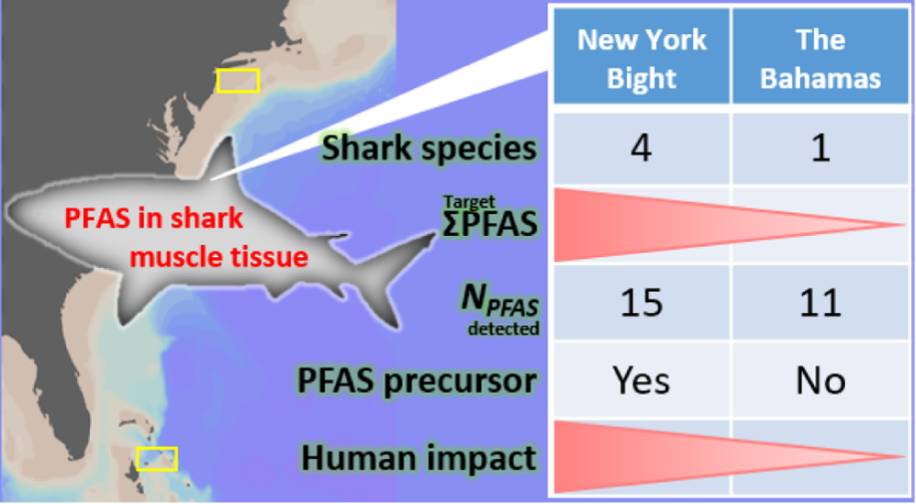

Per- and polyfluoroalkyl substances (PFAS) enter the marine food web, accumulate in organisms, and potentially have adverse effects on predators and consumers of seafood. However, evaluations of PFAS in meso-to-apex predators, like sharks, are scarce. This study investigated PFAS occurrence in five shark species from two marine ecosystems with contrasting relative human population densities, the New York Bight (NYB) and the coastal waters of The Bahamas archipelago. The total detected PFAS (∑PFAS) concentrations in muscle tissue ranged from 1.10 to 58.5 ng g^–1^ wet weight, and perfluorocarboxylic acids (PFCAs) were dominant. Fewer PFAS were detected in Caribbean reef sharks (*Carcharhinus perezi*) from The Bahamas, and concentrations of those detected were, on average, ∼79% lower than in the NYB sharks. In the NYB, ∑PFAS concentrations followed: common thresher (*Alopias vulpinus*) > shortfin mako (*Isurus oxyrinchus*) > sandbar (*Carcharhinus plumbeus*) > smooth dogfish (*Mustelus canis*). PFAS precursors/intermediates, such as 2*H*,2*H*,3*H*,3*H*-perfluorodecanoic acid and perfluorooctanesulfonamide, were only detected in the NYB sharks, suggesting higher ambient concentrations and diversity of PFAS sources in this region. Ultralong-chain PFAS (C ≥ 10) were positively correlated with nitrogen isotope values (δ^15^N) and total mercury in some species. Our results provide some of the first baseline information on PFAS concentrations in shark species from the northwest Atlantic Ocean, and correlations between PFAS, stable isotopes, and mercury further contextualize the drivers of PFAS occurrence.

## Abstract 2

This study reports PFAS concentrations in muscle tissue, which are higher and more diverse in sharks collected from the New York Bight compared to The Bahamas, suggesting strong influences from anthropogenic activities.

## 1 Introduction

Per- and polyfluoroalkyl substances (PFAS) are a group of artificial organofluorine compounds in which carbon–hydrogen (C–H) bonds on an alkyl chain are fully or partially replaced by carbon–fluorine (C–F) bonds. PFAS have been synthesized and applied to a variety of manufacturing processes and products since the 1940s because of their valuable characteristics related to resisting heat, oil, stains, grease, and water.^1^ Their use in commercial products and industrial applications (e.g., textile production, semiconductor industry, electroplating, cooking and baking ware, and aqueous film-forming foam) has resulted in high amounts of PFAS leaching into the environment through various routes, including exhaust gases, wastewater discharge, solid waste, and landfill leachate.^1−5^ Due to the high bond dissociation energy of the C–F bond, PFAS cannot easily be broken down by conventional treatment methods and natural attenuation. Thus, PFAS are widespread and persistent in the environment and are detected in water, air, soil, and wildlife globally, even in remote areas (e.g., the Arctic and deep ocean), due to extensive transport via the hydrological cycle and atmospheric circulation.^2,3,6^

The first study reporting PFAS concentrations in wildlife, including marine species such as tunas and marine mammals, confirmed the ubiquitous distribution of PFAS worldwide.^7,8^ Over the past two decades, a number of studies have reported on the occurrence of PFAS in seawater and marine biota, including plankton, fish, and marine mammals.^9−14^ Longer chain PFAS are associated with high rates of bioaccumulation,^11,15−17^ driving elevated PFAS concentrations in higher-order and long-lived consumers – this offers a direct exposure route to humans through diet.^18^ Ocean environments are often referred to as the “ultimate sink” of many anthropogenic contaminants, including PFAS. However, knowledge of the marine biogeochemical cycling of PFAS and how they are incorporated into marine biota at higher trophic levels remains primitive.

Sharks are meso-to-apex predators, have diverse functional roles, and are often considered sentinels for evaluating the overall health of marine ecosystems.^19−21^ Due to their potential longevity, slow growth, large size, and high trophic level, the tissues of many shark species have been found to contain relatively high concentrations of diverse persistent organic pollutants (e.g., polychlorinated biphenyls [PCBs], dichloro-diphenyl-trichloroethane [DDT], and methylmercury [MeHg]) owing to prolonged bioaccumulation and biomagnification.^22−26^ However, only a handful of studies have reported PFAS concentrations in sharks (e.g., blue sharks (*Prionace glauca*), tiger sharks (*Galeocerdo cuvier*), bull sharks (*Carcharhinus leucas*), and white sharks (*Carcharodon carcharias*)) sampled from the Mediterranean Sea, Atlantic, and Indian Oceans,^27−31^ which often contain long-chain PFAS. For example, perfluorotridecanoic acid (PFTrDA), perfluoroundecanoic acid (PFUnA), and perfluorooctanesulfonate (PFOS) were predominant compounds in muscle tissue, followed by perfluorotetradecanoic acid (PFTA) and perfluorododecanoic acid (PFDoA).^28,29,31^ On average, total detected concentrations of PFAS (∑PFAS) ranged from 0.14 to 17.9 ng g^–1^ (wet weight, ww; muscle tissue), with the highest concentration detected in angular roughsharks (*Oxynotus centrina*) sampled in the Mediterranean Sea.^31^ In general, ∑PFAS in these sharks accumulated with greater propensity in the gonads, vital organs (e.g., heart and liver), and gills rather than muscle tissue.^31^ Concentrations of ∑PFAS in basking sharks (*Cetorhinus maximus*) also varied among tissue types and were higher in skin and liver than in muscle.^28^ One recent study reported higher ∑PFAS concentrations in blood plasma than in muscle tissue of white sharks in the Northwest Atlantic Ocean,^30^ and another recent study revealed interspecific variation in the ∑PFAS of plasma in four small coastal shark species along the South Atlantic Bight.^32^ While intertissue differences in PFAS are common in sharks, additional drivers of bioaccumulation patterns remain speculative. For example, there is limited evidence for the effects of body size and sex on PFAS concentrations.^28,29,31^ However, PFAS concentrations in blue sharks sampled from the northeast Atlantic Ocean^27^ and the Mediterranean Sea^31^ imply a strong effect of geographic region, presumably due to local anthropogenic inputs and food web structure. Regardless of current trends, more extensive monitoring is needed to construct baselines for understanding PFAS concentrations in sharks.

In this study, we provide baseline information on 40 PFAS (including perfluorocarboxylic acids (PFCAs), perfluorosulfonic acids (PFSAs), and their precursors and replacement compounds) measured in the muscle tissue of five species of shark (common thresher shark (*Alopias vulpinus*), sandbar shark (*Carcharhinus plumbeus*), shortfin mako shark (*Isurus oxyrinchus*), smooth dogfish (*Mustelus canis*), and Caribbean reef shark (*Carcharhinus perezi*)) collected from two distinct marine ecosystems: the New York Bight (NYB) and coastal waters of The Bahamas archipelago. The NYB is a temperate, continental shelf ecosystem that spans coastal waters of Cape May, New Jersey, to Montauk Point, New York. In the summer and fall months, this region serves as a critical habitat for many shark species^33−35^ but is known to receive large influxes of anthropogenic pollutants from the metropolitan area of New York City via river discharge, stormwater runoff, sewage and wastewater treatment discharge, and landfill leachate.^36,37^ The coastal waters of The Bahamas are relatively pristine, comprising a mosaic of habitats, including mangroves, seagrasses, coral reefs, open ocean, and deep-sea environments.^38−40^ This region is known to support high shark biomass and diversity, attributed to decades of minimized anthropogenic disturbance and protection from commercial fishing.^41−44^ Moreover, this region is assumed to receive much lower amounts of land-derived anthropogenic contaminants than the NYB.^25^

Specific objectives were to compare PFAS occurrence and relative abundance among five shark species from the two distinct study sites and examine correlations between PFAS across individuals. Furthermore, we explored whether relationships exist between PFAS concentrations and nitrogen stable isotope values (δ^15^N) and total mercury (THg), which can serve as proxies for relative trophic position.^45,46^ Finally, the isomeric composition of PFOS was examined to evaluate the fractionation between linear and branched PFOS in different shark species. These findings provide baseline information on PFAS in multiple shark species from the northwestern Atlantic and combine concentrations with complementary chemical proxies to contextualize drivers of bioaccumulation.

## 2 Materials and Methods

### 2.1 Sample Collection

#### 2.1.1 New York Bight

Four shark species were investigated from the NYB, including common thresher shark (*n* = 8, fork length (FL): 77–190 cm, male = 5, female = 3), sandbar shark (*n* = 17, FL: 104–144 cm, male = 10, female = 7), shortfin mako shark (*n* = 8, FL: 121–212 cm, male = 6, female = 2), and smooth dogfish (*n* = 11, FL: 52–104 cm, male = 4, female = 6) (Table S1). All research was conducted under research permits granted by the New York State Department of Environmental Conservation (NYSDEC). Shortfin mako, sandbar shark, and common thresher were captured using traditional rod and reel angling throughout the south shore of Long Island, New York (Figure S1), during the summer of 2017 and 2018. Upon capture, animals were restrained alongside the research vessel prior to sampling. Smooth dogfish were opportunistically sampled during inshore benthic trawl surveys conducted by Stony Brook University on behalf of NYSDEC and, upon capture, were placed in an aerated holding tank for approximately 1 h until sampling. Approximately 1 g of dorsal white muscle tissue was excised from the dorsal musculature using a customized melon baller and placed on ice. The adopted melon baller works just as a biopsy punch would, but to better effect, and has been reported in studies elsewhere.^47^ Animals were then measured (precaudal length, fork length, and total length), sexed, and subsequently released. All tissue samples were stored at −20 °C upon return to the laboratory until further processing.

#### 2.1.2 Coastal Bahamas

One shark species, the Caribbean reef shark (*n* = 18, FL: 105–160 cm, male = 3, female = 7), was studied from The Bahamas (Table S1). All research was conducted between January 2018 and March 2020 under research permits granted by The Bahamas Ministry of Agriculture and Marine Resources, Department of Marine Resources (MAMR/FIS/17&34^A^, MA&MR/FIS/9, and MAMR/FIS/2/12*A*/17/17B). Sharks were captured using experimental scientific drumlines^48^ and longlines^41^ from two primary locations: Nassau, New Providence (24.977°N, −77.501°W), Great Exuma (23.727°N, −76.034°W) and Cape Eleuthera (24.833°N, −76.354°W) (Figure S1). Upon capture, animals were secured alongside the research vessel, and Bahamian sharks were sampled similarly to those from New York. Sharks were then sexed and measured as above prior to release. Upon return to the laboratory, all tissue samples were stored at −20 °C until further processing.

### 2.2 PFAS Analysis

All chemicals and solvents used in this study were certified ACS reagent grade and LC/MS grade with high purity and were purchased from Sigma-Aldrich (Burlington, MA), Honeywell (Charlotte, NC), and Fisher Scientific (Pittsburgh, PA). PFAS native and isotopically labeled standards were purchased from Wellington Laboratories (Guelph, ON). The abbreviation of all studied compounds is summarized in Table S2.

The method for tissue extraction was modified from EPA draft method 1633. About 100 mg of freeze-dried muscle tissue was transferred into a 15 mL polypropylene (PP) centrifuge tube. A suite of isotopically labeled PFAS surrogate standards (SUR, 0.5–10 ng each, see Table S2) was spiked directly into each tube. The extraction procedure involved three steps. First, 10 mL of 0.05 mol L^–1^ potassium hydroxide (KOH, ACS grade) in methanol (MeOH, LC–MS grade) was added into each sample, mixing the tissue sample and swirling it on a mixing table to extract for 16 h. The supernatant was collected in a 50 mL PP centrifuge tube after centrifugation (2800 rpm, 10 min). Second, 10 mL of acetonitrile (ACN, LC–MS grade) was added into the remaining tissue in the 15 mL PP tube, sonicating it for 30 min. After centrifugation at 2800 rpm for 10 min, the supernatant was transferred into the 50 mL PP tube containing the first extract. Third, 5 mL of 0.05 mol L^–1^ KOH in MeOH was added to the remaining sample, vortexing to disperse the tissue. After centrifugation at 2800 rpm for 10 min, the supernatant was collected and combined with the first two extracts in the 50 mL PP tube. The combined extract (∼25 mL) was evaporated to ∼2.5 mL under a smooth stream of nitrogen flow. Ultrapure water (Milli-Q water, 18.2 MΩ·cm, Millipore Milli-Q Integral 5 water purification system) was added to each tube to dilute the concentrated extract to 50 mL.

Solid phase extraction (SPE) was performed with a weak anion exchange (WAX) cartridge (Waters Oasis WAX, 150 mg, 6 mL) to clean up the extracts. A 24-position vacuum manifold was set up for SPE. Each SPE cartridge was conditioned with 15 mL of 1% methanolic ammonium hydroxide (NH_4_OH, ACS grade) followed by 5 mL of 0.3 mol L^–1^ formic acid (LC–MS grade). The sample was then transferred into the reservoir on top of the SPE cartridge, passing the entire sample through the cartridge at 5 mL min^–1^ and rinsing the reservoir wall with ultrapure water afterward. The cartridge was dried by vacuum for 5 min. The empty 50 mL sample tube was rinsed with 2.5 mL of 1% methanolic NH_4_OH, and the SPE cartridge was eluted with the rinsate to collect the SPE extract in a collection tube (15 mL PP tube). This rinsing/elution step was performed twice. The final ∼5 mL SPE extract was then blown down to near dryness and reconstituted to 1 mL with MeOH. A suite of isotopically labeled PFAS internal standards (IS, 1 ng each, see Table S2) was added to achieve a final concentration of 1 ng mL^–1^. This final extract was stored at 4 °C prior to analysis.

An Agilent 6495B liquid chromatography–tandem mass spectrometry (LC–MS/MS) system with an ESI (electrospray ionization) source was employed for PFAS analysis. In brief, the LC system consists of a binary pump, multisampler, and column thermostat. A C18 analytical column (3.0 × 50 mm, 1.8 μm) is used for separation, and a delay column (C18, 4.6 × 50 mm, 3.5 μm) is installed between the pump and multisampler to prevent any background PFAS (present in the mobile phases or the LC pump units) from eluting within the retention time windows of the target PFAS. The column temperature was 50 °C, and the injection volume was 5 μL. The mobile phases are (A) 5 mmol L^–1^ ammonium acetate in water and (B) MeOH with a flow rate of 0.4 mL min^–1^. The MS system was operated in negative mode, and the detailed LC and MS parameters are listed in Table S3. The multiple reaction monitoring (MRM) transitions for 40 native PFAS and their corresponding labeled isotopes are listed in Table S2. The acquired data were processed by Agilent MassHunter Qualitative (B.08.00) and Quantitative (B.09.00) Analysis software to calculate the concentration corrected by SUR and IS. All PFAS concentrations reported in this study are presented on a wet weight (ww) basis if not otherwise noted.

For QA/QC purposes, multiple procedural and spiked blanks were prepared in every extraction batch. The estimated method detection limits (MDLs) for each analyte are summarized in Table S2. A certified reference material (CRM), IRMM-427 (PFAS in fish tissue), was used to go through the above extraction procedure to evaluate the analytical accuracy. Due to the limited tissue mass, sample duplicate and matrix spike samples were not performed. The QA/QC results, including CRM results and surrogate isotope recoveries, are summarized in Table S4. The measured PFAS concentrations in the CRM samples were within the certified values, except for PFHxS. PFHxS seemed to be influenced severely by matrix interference with our chromatographic condition, so PFHxS was excluded. For all PFAS analytes, we monitored at least two MRM transitions (if possible) to ensure confidence in the quantitation. Perfluorobutanoic acid (PFBA) and perfluoropentanoic acid (PFPeA) both have only one available MRM for identification and quantification. Matrix interference can occur while measuring these two PFAS in biota samples with a low-resolution LC–MS/MS, resulting in false positive results.^49,50^ Therefore, the concentrations of PFBA and PFPeA in this study were reported as indicative values with no further discussion.

### 2.3 Stable Isotope Analysis (SIA) and Total Mercury (THg)

For a subset of individuals, muscle tissue was analyzed for carbon and nitrogen stable isotopes. Muscle tissue was dried and ground to a fine powder using a mortar and pestle before being triple rinsed with DI water to remove ^14^N-enriched nitrogenous compounds (e.g., urea and trimethylamine N-oxide).^51−53^ Samples were then redried, and 0.25–0.35 mg of tissue was weighed into 5 × 7 mm tin capsules. Samples were combusted on a Thermo Delta V plus continuous flow isotope ratio mass spectrometer (Bremen, Germany) coupled to a flash elemental analyzer (EA-IRMS). Carbon and nitrogen stable isotope values are reported in delta notation (δ) relative to Vienna Pee Dee Belemnite (V-PDB) for δ^13^C and air for δ^15^N. Instrument drift and precision were evaluated by repeat runs of internationally certified reference standards of glycine (USGS65), IU l-glutamic acid, and caffeine (IAEA-600), for which precision (standard error) did not exceed 0.02 and 0.06 for δ^13^C and δ^15^N, respectively. All stable isotope analyses were conducted in the Department of Geosciences at Stony Brook University, USA.

Direct measurement of THg in dried muscle tissue was conducted by a Milestone Direct Mercury Analyzer (DMA-80). The detailed analytical procedure and the data were published elsewhere.^25^ THg was acquired only for Caribbean reef sharks and was not available for NYB shark species.

### 2.4 Data Analysis

The total PFAS (∑PFAS) concentration is defined as the summary of all detected PFAS concentrations. In addition to performing data analysis on single PFAS and ∑PFAS concentrations, we also categorized them into three groups: short-chain (C ≤ 6), long-chain (7 ≤ C ≤ 9), and ultralong-chain (C ≥ 10) PFAS because the carbon chain length significantly controls PFAS physical and chemical characteristics. The Mann–Whitney U and Kruskal–Wallis tests were used to test for differences in ∑PFAS concentrations between two or more data groups (e.g., shark species, habitats, sex, and length). The Pearson correlation test was used to assess relationships between individual PFAS compounds and relationships between PFAS and other variables like fork length, stable isotopes, and THg, if values were available for comparison. Positive and negative coefficients denote positive and negative correlations, respectively. The strength of the correlation is adopted as follows: 0.0–0.1 (negligible), 0.1–0.39 (weak), 0.40–0.69 (moderate), 0.70–0.89 (strong), and 0.90–1.00 (very strong).^54,55^ All data analyses were performed in Microsoft Excel and R (version 4.2.3) with a significance level α set at 0.05.

## 3 Results and Discussion

### 3.1 PFAS Occurrence in Shark Tissues

**Figure 1:**
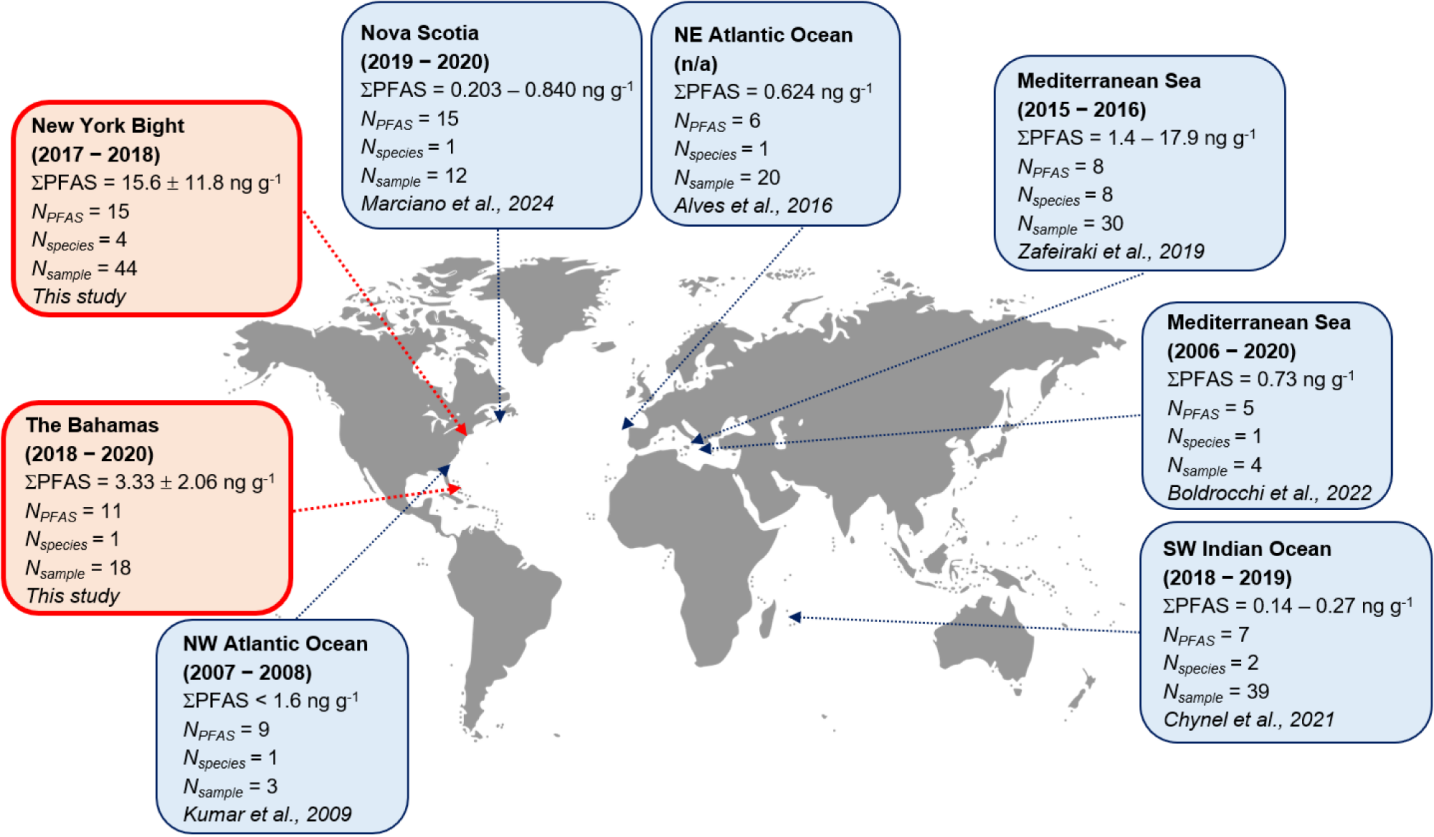
Reported PFAS concentrations (ng g^–1^, wet weight) in muscle tissue of marine sharks in previous literature and in this study. The year in the parentheses represents the sampling year. ΣPFAS = the sum of detected PFAS concentrations; *N*_PFAS_ = the number of PFAS detected; *N*_species_ = the number of shark species investigated; *N*_sample_ = the total number of individuals analyzed.

Total detected PFAS (∑PFAS) ranged from 1.10 to 58.5 ng g^–1^ (mean: 12.0 ng g^–1^). The mean concentration aligns with previously reported values of muscle tissue for cartilaginous fishes^11^ (Figure 1 and Table S5). Our measured ∑PFAS was similar to sharks in the coastal Mediterranean.^31^ In contrast, ∑PFAS in sharks caught in open oceans (e.g., Atlantic and Indian Oceans) was <1 ng g^–1^,^27−29^ more than an order of magnitude lower than sharks in the Mediterranean Sea and those analyzed in this study. It should be noted that ∑PFAS depends on the amount of PFAS analyzed and detected. Due to the increasing commercial availability of PFAS calibration standards, improved sample preparation, and instrumentation, more PFAS can be confidently determined. Therefore, the total number of PFAS detected in this study was nearly double that of most previous shark studies, which resulted in elevated ∑PFAS.

**Figure 2:**
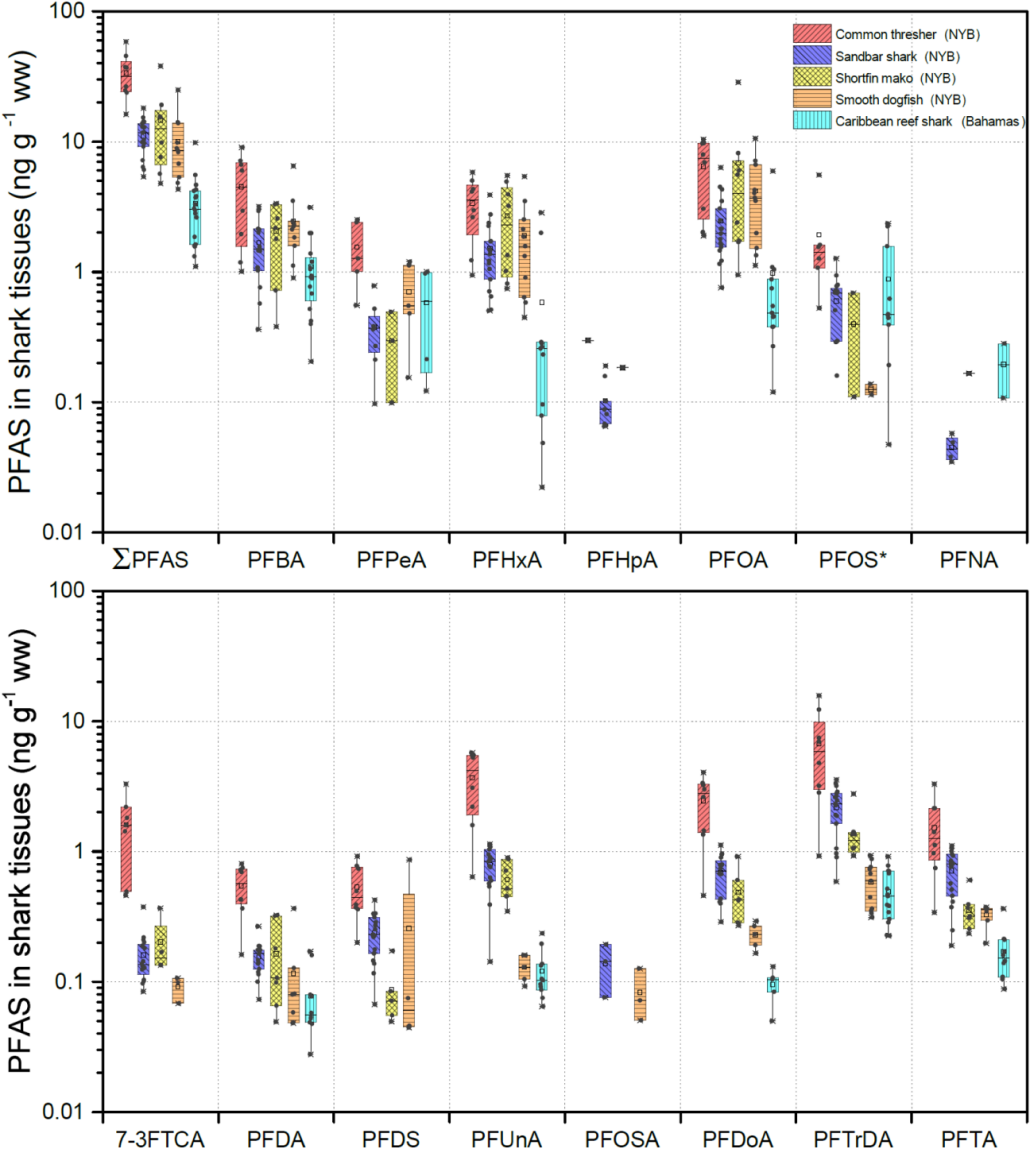
PFAS concentrations in muscle tissue of five shark species collected from the New York Bight and the coastal Bahamas. PFOS* = linear PFOS + branched PFOS. The upper edge, middle line, and lower edge of the box represent the upper quartile (75%), median (50%), and lower quartile (25%) of the data, respectively. The open square represents the mean value. The upper and lower whiskers represent the 1.5× interquartile range (IQR), and the data beyond the 1.5× IQR are considered the outliers (times symbol).

A total of 15 targeted PFAS, including PFCA, PFSA, polyfluorotelomer, and perfluorosulfonamide, were detected in the tissues of the five focal species (Figure 2 and Table S5). PFTrDA (95%), PFOA (92%), PFBA (92%), and PFHxA (89%) were the four most frequently detected compounds, whereas PFOSA (10%), PFNA (11%), and PFHpA (18%) were detected at the lowest frequency. Among the 15 detected PFAS, PFOA (mean: 3.63 ng g^–1^; range: 0.12–28.6 ng g^–1^) was the most abundant, followed by PFBA (mean: 2.10 ng g^–1^; range: 0.21–9.05 ng g^–1^), PFTrDA (mean: 1.96 ng g^–1^; range: 0.23–15.8 ng g^–1^), and PFHxA (mean: 1.85 ng g^–1^; range: 0.020–5.82 ng g^–1^). For ultralong-chain PFCAs (i.e., C ≥ 10), those containing an odd number of carbon atoms have been found to dominate fish tissues.^17,29,56−58^ Indeed, PFTrDA and PFUnA (which contain an odd number of carbon atoms) were more abundant in shark muscle than PFDoA and PFTA (which contain an even number of carbon atoms) in this study. This might be attributed to atmospheric degradation of PFCA precursors (e.g., fluorotelomer alcohol, FTOH) released into the environment.^59^

For both individual species and pooled species, we found no significant effect of sex on PFAS concentrations (Table S6), which aligns with previous studies on several shark species (e.g., blue shark, bluntnose sixgill shark (*Hexanchus griseus*), and sharpnose sevengill shark (*Heptranchias perlo*)).^27,31^ Most relationships were not significant between fork length and individual PFAS, ∑PFAS, and long-chain and ultralong-chain PFAS in muscle tissue (Table S7). Two exceptions were PFDoA in common thresher sharks and PFDS in sandbar sharks, which exhibited significant negative (*r* = −0.718, *p* = 0.045) and positive (*r* = 0.490, *p* = 0.046) correlations, respectively. A more extensive measurement of PFAS in sharks covering a wide range of length and weight with representative sample sizes would help confirm whether PFAS in marine fish accumulate like other pollutants or behave uniquely.

### 3.2 Correlations between Individual PFAS in Muscle Tissue

**Figure 3:**
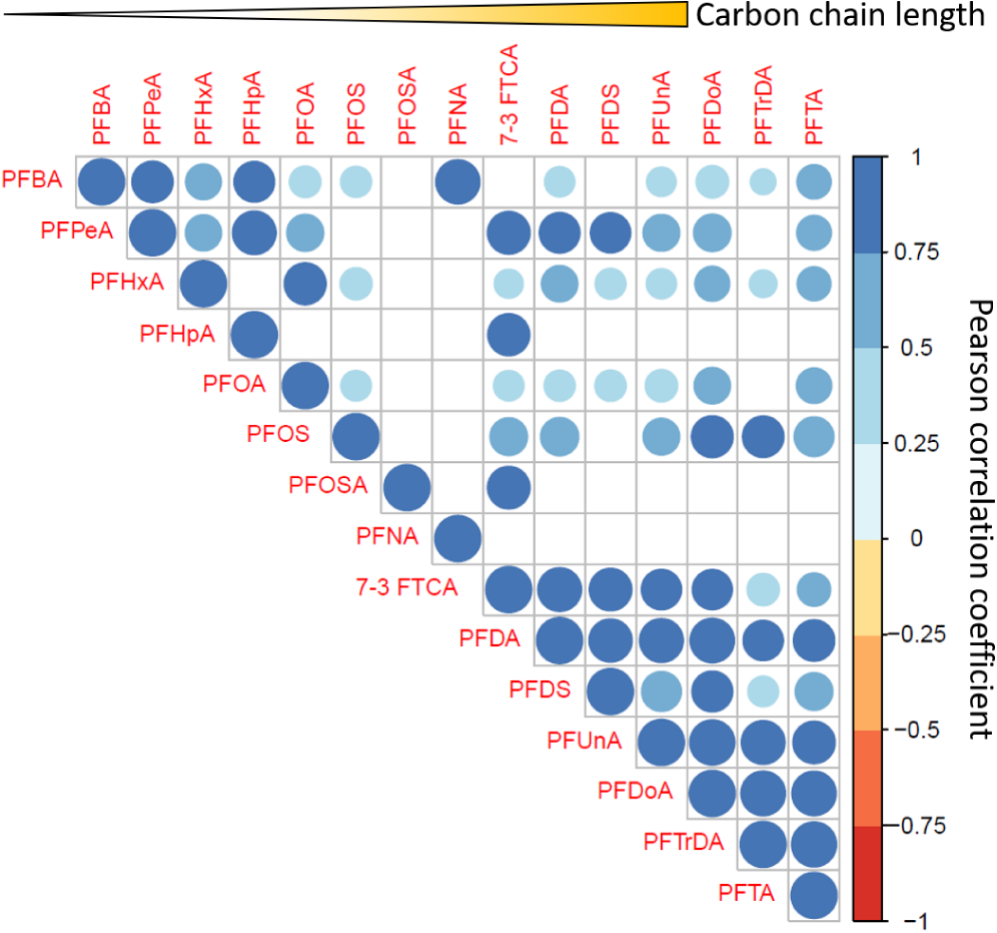
Correlogram of Pearson’s correlations between PFAS in all shark muscle tissues analyzed in this study. Only significant correlations (*p* < 0.05) are shown in the plot. The carbon chain lengths are in order from shortest to longest from left to right along the top of the figure. Larger circles represent larger correlation coefficients and the blue and red color ramps indicate positive and negative correlations, respectively.

Using pooled data from all sharks, we compared correlations between any two PFAS measured in muscle tissue (Figure 3). Most PFAS exhibited significant positive correlations, except for those with low detection frequency (e.g., PFHpA, PFNA, PFOSA), for which no significant correlation was found. Interestingly, strong positive correlations (Pearson’s *r* > 0.75) were observed among the ultralong-chain PFAS. This result may imply that sharks could acquire each existing PFAS (regardless of the chain length) from the environment, and the PFAS equilibrium present in tissue may differ between compounds, presumably due to the greater elimination rate of short-chain PFAS in organisms.^60−62^ Routes of PFAS exposure to sharks could be another critical factor. For example, ultralong-chain PFAS were likely acquired via diet because of their greater affinity for interacting with biotic/abiotic suspended particles, resulting in frequent detection in marine organisms, such as plankton,^9,14,63^ but rare detection in seawater.^11,12,64^ The uptake of PFAS with a relatively shorter chain length (e.g., PFOA) could be absorbed directly from either ambient water or diet. For instance, PFOA uptake by rainbow trout (*Oncorhynchus mykiss*) showed a moderate dietary assimilation efficiency (59 ± 5.0%) and aqueous uptake rate (0.53 ± 0.041 L kg^–1^ d^–1^).^60,61^

### 3.3 Comparisons between Species and Habitats

Among five different shark species, mean ∑PFAS concentrations were highest in common thresher sharks (33.7 ng g^–1^) > shortfin mako sharks (14.5 ng g^–1^) > sandbar sharks (11.1 ng g^–1^) > smooth dogfish (10.0 ng g^–1^) > Caribbean reef sharks (3.33 ng g^–1^) (Figure 2 and Table S5). Shortfin mako sharks, sandbar sharks, and smooth dogfish did not have significantly different ∑PFAS (*H* = 2.188, *p* = 0.33). The ∑PFAS concentrations (especially ultralong-chain PFAS) of common thresher sharks were notably higher than any other species from the NYB, which may be directly related to their feeding habits and trophic level. Common thresher sharks primarily feed on bony fish, exhibiting narrower dietary preferences in comparison with other shark species,^65,66^ whereas shortfin mako sharks, sandbar sharks, and smooth dogfish feed across multiple trophic levels with diets comprising bony fish, mollusks, and crustaceans.^65,67−70^ Nevertheless, common thresher sharks did not exhibit the highest δ^15^N values among the studied NYB species (Figure S2), suggesting that trophic position may not be the primary factor contributing to their high ∑PFAS concentrations in this study. Other factors, such as sexual maturity, thermoregulation, migration patterns, and habitat associations, may also drive variation in PFAS concentrations. For instance, common thresher and shortfin mako sharks are regionally endothermic and have higher mean ∑PFAS than others in this study. Endotherms may have higher energy demands and lower food conversion efficiencies than ectotherms, resulting in greater intake and accumulation of contaminants.^71,72^ Notably, most of the common thresher sharks analyzed in this study were immature,^73^ suggesting that high ∑PFAS concentrations could also be attributed to maternal transfer.^29^ The subsequent growth to maturity may induce growth dilution to lower PFAS concentrations in the muscle tissue. There was only one tissue sample from a mature common thresher shark in this study, which exhibited much lower ultralong-chain PFAS concentrations (3.22 ng g^–1^, *n* = 1) than the mean of the immature conspecifics (18.9 ± 7.5 ng g^–1^, *n* = 7). Migration patterns differ between shark species, and bioaccumulative contaminants in sharks reflect a sum of spatial and temporal integration. Recent studies revealed that Caribbean reef sharks show little evidence of seasonal migration, and thus, they can be considered a migration control group for those captured from the NYB.^74,75^ Common thresher sharks, sandbar sharks, and smooth dogfish are highly migratory along the coastal, northwestern Atlantic, whereas shortfin mako sharks exhibit a wider migratory range across the Atlantic Ocean.^76^ In short, PFAS accumulation in these sharks could be driven by a combination of multiple biological and environmental factors acting at the species level.

**Figure 4:**
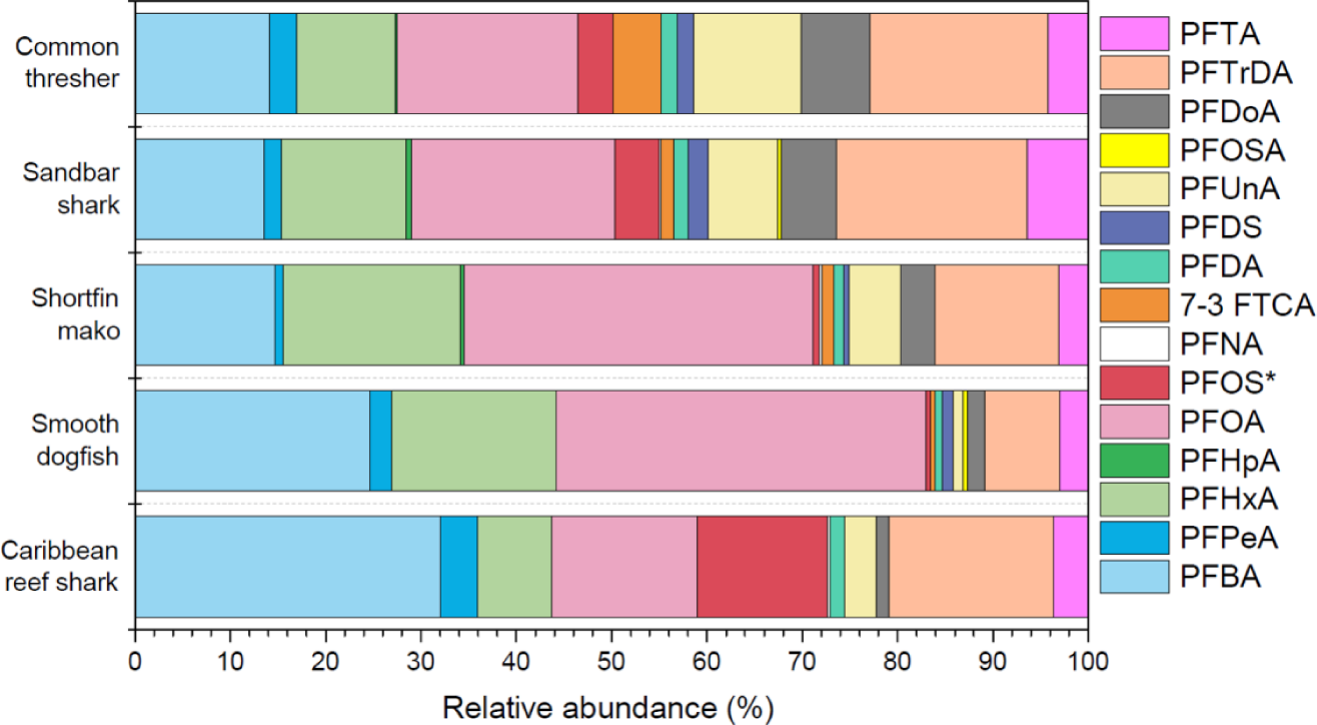
Relative abundance of PFAS in muscle issue of five shark species in this study. PFOS* = total PFOS (linear + branched).

Fewer compounds at lower concentrations were detected in Caribbean reef sharks (*n* = 11) than the species sampled from the NYB (*n* = 13–15 per species) (Figure 2). Although ∑PFAS concentrations varied in the four NYB shark species, the ratio of the dominant PFAS compounds did not fluctuate drastically and followed a sequence of PFOA > PFTrDA ≈ PFHxA ≈ PFBA (Figure 4), and PFCA and PFSA accounted for >90% and <7% of ∑PFAS, respectively. In contrast, PFSA comprised only 14% of ∑PFAS (PFCA = 86%) in the Caribbean reef sharks, and the PFOS concentration was comparable to PFOA and PFTrDA. Lastly, PFDS, PFHpA, and PFAS precursors, such as 7:3 FTCA and PFOSA, were only detected in the NYB sharks. These results suggest divergent PFAS source(s) between The Bahamas and coastal New York. The NYB marine ecosystem is susceptible to a large input of anthropogenic materials from the tristate metropolitan area and exhibits benthic-pelagic coupling like many expansive continental shelf ecosystems.^77,78^ These could explain higher accumulation of more diverse PFAS and their precursors in sharks from the NYB than those from The Bahamas. One study surveyed PFAS concentrations in seawater and plankton in the continental shelf of the northwestern Atlantic, including the NYB.^14^ Unfortunately, 7:3 FTCA was not measured, and PFOSA was below the detection limit. However, PFOSA along with other precursors such as 6:2 fluorotelomer sulfonic acid (6:2 FTSA) and *N*-ethylperfluorooctane sulfonamidoacetic acid (NEtFOSAA) was consistently detected in plankton samples, suggesting PFAS precursors’ occurrence and availability to enter biota at the base of marine food webs in the NYB.^14^ Thus, we suspect that the accumulation of PFAS precursors and their transformation to end products like PFCA and PFSA may occur as PFAS were transferred and propagated through subsequent trophic levels. Further investigation is required to survey PFAS occurrence and distribution in the water column, sediment, and biotas (e.g., plankton, forage fish, and benthos) in the NYB.

### 3.4 δ^13^C and δ^15^N Stable Isotope Ratios versus PFAS

**Figure 5:**
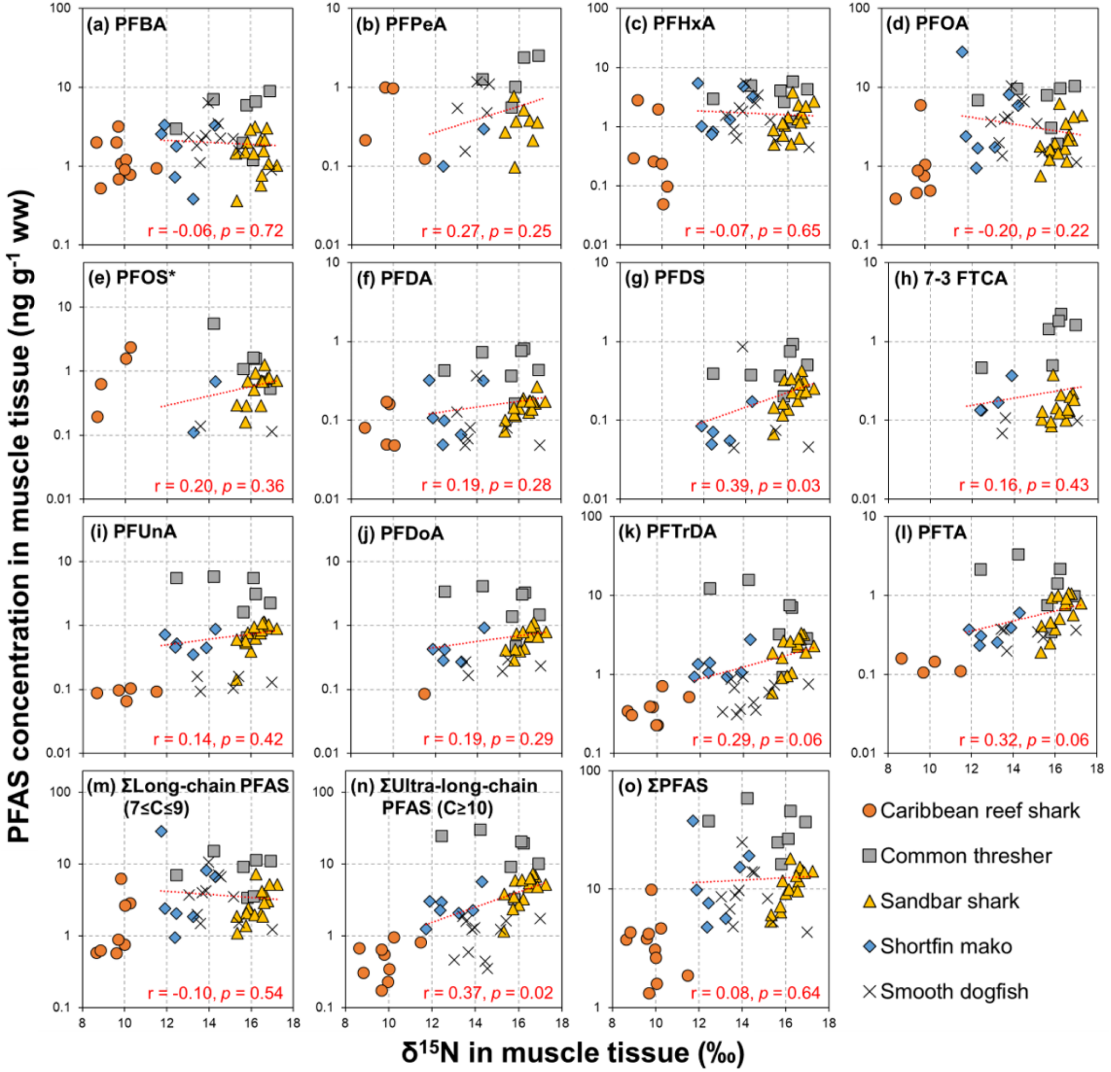
PFAS concentrations as a function of δ^15^N values in muscle tissue of five shark species sampled from The Bahamas and the NYB. Red dotted lines denote a correlation line calculated solely from all the NYB shark species (i.e., the Caribbean reef shark was excluded). The calculated correlation coefficient and *p*-value are reported in each panel.

Correlation coefficients between stable isotope values and PFAS concentrations in the muscle tissue of five shark species are summarized in Tables S8 and S9. We acknowledge that cross-system comparisons of δ^13^C and δ^15^N can be confounded by varying isotope baselines.^79^ Therefore, statistical analysis for pooled species only included samples caught in the NYB, and δ^15^N values of the Caribbean reef shark are provided in Figure 5 for visual comparison only. In general, δ^13^C values in sharks (single or pooled species) were rarely correlated with any individual PFAS or ΣPFAS. In contrast, we found significant positive correlations between δ^15^N and individual PFAS, especially in sandbar sharks. For other species, we observed sporadic positive significant correlations between δ^15^N and PFAS.

Most PFAS (C ≥ 6) in sandbar sharks revealed a significant and moderate positive correlation (*r* > 0.5 and *p* < 0.05) with δ^15^N (Table S9), suggesting that PFAS accumulation is likely to be associated with diet across ontogeny. Among all ultralong-chain PFAS detected in sandbar sharks, only 7:3 FTCA showed no significant relationship with δ^15^N. The result may indicate that 7:3 FTCA in sharks was not primarily assimilated from the surroundings but rather an intermediate metabolite during the degradation from 8:2 fluorotelomer alcohol (8:2 FTOH) or 8:2 fluorotelomer phosphate diester (8:2 diPAP) to PFCA.^80 −83^ Also, 7:3 FTCA may further undergo metabolic transformation to form PFHpA or PFOA inside sharks;^80,83^ therefore, the accumulation signal of 7:3 FTCA might be diluted. Linear PFOS concentrations in sandbar sharks were significantly correlated with δ^15^N. However, the branched PFOS showed no correlations, suggesting different bioaccumulation dynamics for branched PFOS, which are vulnerable to faster metabolic depuration *in vivo*. Pooled species analysis of the NYB sharks showed weaker positive correlations with δ^15^N for PFOS and all ultralong-chain PFAS (C ≥ 10) (Figure 5 and Table S9), although only PFDS and ΣUltralong-chain PFAS were statistically significant (Table S9). Substantially higher PFAS concentrations in common thresher sharks than the other species may complicate the trends and statistical results of the cross-species analysis.

Only a few studies have reported PFAS concentrations associated with nitrogen stable isotope data in a single marine animal. For example, a significant positive correlation was reported between δ^15^N values of muscle tissue and PFOS concentrations in the livers of marine mammals in the North Sea^84^ and polar cod (*Boreogadus saida*) in the Barents Sea.^85^ Their results agreed with our observation of PFAS in sandbar sharks, although our PFAS measurement came from muscle tissue. Unfortunately, we did not measure PFAS concentrations and corresponding δ^15^N values across the entire food webs of the two study locations, so we could not cover a broader range of trophic levels to evaluate potential PFAS biomagnification in sharks.

### 3.5 PFAS Isomers and Total Mercury Concentrations (THg)

**Figure 6:**
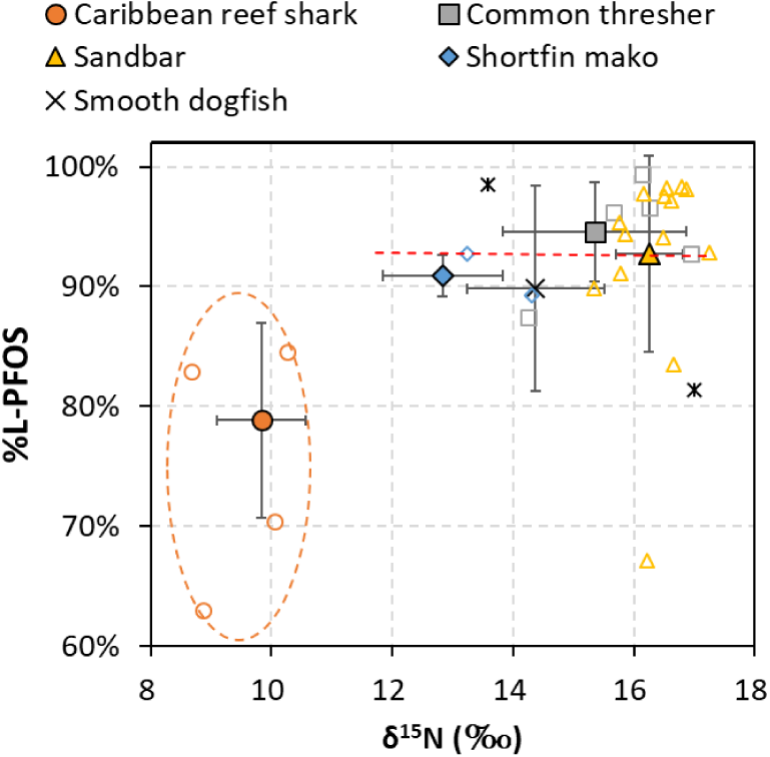
Percentage of linear PFOS (%L-PFOS) in five shark species as a function of δ^15^N in muscle tissue. The filled markers represent the average and standard deviation, and the open markers indicate the actual data distribution. Data within the orange dashed circle represent sharks from The Bahamas.

Isomeric fractionation of PFAS has been observed in the environment, leading to a shift of linear-to-branched ratios in various environmental compartments.^86,87^ In this study, the percentage of linear PFOS (%L-PFOS) was 79 ± 8% (*n* = 10) in Caribbean reef sharks and 93 ± 7% (*n* = 25) in the NYB sharks, highlighting another distinct difference between these two habitats. One explanation could be the origin of PFOS in the two ecosystems, in which the electrochemical fluorination process produced PFOS with a ratio of about 70:30 linear:branched, and the telomerization process produced ∼100% linear PFOS.^86^ The isomeric composition can also be altered during wastewater treatment processes before discharge.^87^ Moreover, biogeochemical processes such as particle scavenging and algal uptake could also enrich L-PFOS from the aqueous phase onto the particulate phase.^88^ Subsequent bioaccumulation and trophic transfer may continuously fractionate PFAS isomers because branched PFOS is more accessible to eliminate and excrete, as shown in animal experiments.^89,90^Figure 6 shows relationships between %L-PFOS and δ^15^N values. Among the NYB sharks, no relationship was found between %L-PFOS and δ^15^N values. The signature of high %L-PFOS might directly originate from the sharks’ diet and the subsequent isomeric fractionation through *in vivo* metabolism.

**Figure 7:**
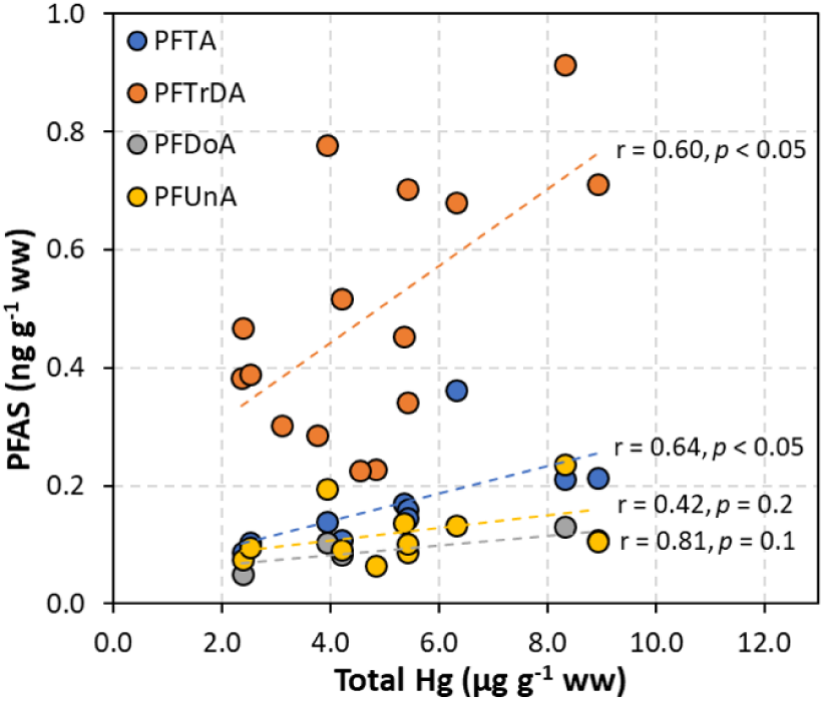
Correlations between ultralong-chain PFAS and total Hg in muscle tissue of the Caribbean reef shark (*C. perezi*).

The presence of PFAS in fish with other contaminants, such as mercury (Hg), is of interest due to food safety concerns.^28,91,92^ Total mercury (THg) concentrations of the Caribbean reef sharks in this study have been published elsewhere,^25^ ranging from 2.4 to 8.9 μg g^–1^ (mean = 4.5 ± 1.9 μg g^–1^, *n* = 18). Methylmercury (MeHg) is the dominant Hg species in fish because it bioaccumulates readily via dietary exposure.^93^ As expected, THg revealed positive, moderate correlations with many ultralong-chain PFAS like PFTA, PFTrDA, PFDoA, and PFUnA (Figure 7), although the linear relationships for the latter two compounds (i.e., PFDoA and PFUnA) were not statistically significant. Very few studies have compared THg and PFAS concentrations in vertebrates, with existing data limited to freshwater fish. For example, one study reported significant positive correlations between THg and C9–C15 PFCA in liver tissue of four trout species from high-mountain lakes in France.^94^ Another found that THg in muscle tissue was positively correlated with PFOS and PFUnA in Nile Perch (*Lates niloticus*) from northern Lake Victoria, East Africa.^95^ Their results aligned with this study, implying similar exposure sources and accumulation pathways of THg and some PFAS (e.g., ultralong-chain) in fish.

### 3.6 Public Health Implications

Consumption of shark meat as a human protein source has been growing globally,^96^ and given that PFAS are ubiquitous in the environment and could have adverse health effects on humans via seafood consumption,^18^ there is a need to establish a baseline of PFAS in sharks. For example, the European Food Safety Authority (EFSA) currently established a group tolerable weekly intake (TWI) of 4.4 ng kg^–1^ body weight per week for the sum of PFOS, PFOA, PFNA, and PFHxS.^97^ Meanwhile, the maximum levels (MLs) for PFAS in fish muscle meat were set to 2.0–35 (PFOS), 0.2–8 (PFOA), 0.5–8 (PFNA), and 0.2–1.5 (PFHxS) ng g^–1^, depending on fish species (no sharks included).^98^ About 2% (PFOA), 91% (PFOS), and 100% (PFNA) of the muscle tissue samples in this study were below the lower bound MLs set for each PFAS. A small portion of the samples (11%) exceeded the upper bound ML (8 ng g^–1^) set for PFOA, whereas PFOS and PFNA were all below the upper MLs. If we assume 8 oz (∼226.8 g)^99^ of shark meat consumption once a week and a body weight of 70 kg, 84% of the muscle tissue samples exceeded EFSA’s TWI (for the sum of PFOA, PFOS, and PFNA). Advisories and regulations issued by governments toward PFAS concentrations in various environmental compartments have been rising with increasingly available data on PFAS measurements. Our data can serve as a baseline for policy-making against PFAS contamination in these two critical marine ecosystems and seafood safety for human exposure.

Our findings are important from a consumption perspective, and our results provide evidence of multiple PFAS presented in five shark species collected from the NYB and the coastal waters of The Bahamas. Sharks captured in the NYB may be impacted, to a greater extent, by anthropogenic inputs relative to those from The Bahamas, highlighting the significance of local contamination sources for PFAS exposure. We also demonstrated positive correlations between ultralong-chain PFAS and other chemical indicators, such as δ^15^N and total mercury in some shark species, suggesting potential dietary drivers of bioaccumulation. Compared to traditional pollutants, PFAS seem to behave differently when accumulating in fish. Thus, extensive and comprehensive monitoring of PFAS in sharks is necessary to explore the role of biological and environmental factors in PFAS accumulation in these important meso-to-apex predators.

## Supporting Information Available

Additional details on sampling locations, analytical methods, instrumental conditions, and QA/QC; summary of PFAS concentrations in shark muscle tissue in the current and previous studies; summary of statistical significance or correlations between PFAS and sex, length, and stable isotopes (PDF)
