# Supplementary material for: Accumulation of Per- and Polyfluoroalkyl Substances (PFAS) in Coastal Sharks from Contrasting Marine Environments: The New York Bight and The Bahamas
Source: Environ Sci Technol. 2024 Jul 12;58(29):13087–98. doi: 10.1021/acs.est.4c02044 (PMC11270988; doi:10.1021/acs.est.4c02044)
Supplement: Supplementary file 1 — es4c02044_si_001.pdf [file es4c02044_si_001.pdf]

Supporting Information for

Accumulation of per- and polyfluoroalkyl substances (PFAS) in coastal sharks from contrasting marine environments: The New York Bight and The Bahamas

Cheng-Shiuan Lee[1,*], Oliver N. Shipley[2], Xiayan Ye[3], Nicholas S. Fisher[2], Austin J. Gallagher[4], Michael G. Frisk[2], Brendan S. Talwar[5], Eric V.C. Schneider[5], Arjun K. Venkatesan[6]

[1] Research Center for Environmental Changes, Academia Sinica, Taipei 115, Taiwan

[2] School of Marine and Atmospheric Sciences, Stony Brook University, Stony Brook, NY 11790, USA

[3] New York State Center for Clean Water Technology, Stony Brook University, Stony Brook, NY 11794, USA

[4] Beneath the Waves, Boston, MA, 02129, USA

[5] Cape Eleuthera Institute, Rock Sound, Eleuthera, 26029, The Bahamas

[6] Department of Civil and Environmental Engineering, New Jersey Institute of Technology, Newark, NJ 07102, USA

*Corresponding Author: Cheng-Shiuan Lee, Phone: +886 2 7875868, chenglee@gate.sinica.edu.tw

This file contains:

Pages S1 to S14

Tables S1 to S9

Figures S1 to S2

Table S1. Information of shark species studied

#	Location	Species	Common name	n	Fork length (cm)	Sex	Immature individuals
1	The Bahamas	*Carcharhinus perezi*	Caribbean reef shark	18	105 − 160	M = 3, F = 7	0
2	New York Bight	*Alopias vulpinus*	Common thresher	8	77 − 190	M = 5, F = 3	7
3	New York Bight	*Carcharhinus plumbeus*	Sandbar shark	17	101 − 144	M = 10, F = 7	11
4	New York Bight	*Isurus oxyrinchus*	Shortfin mako	8	121 − 212	M = 6, F = 2	8
5	New York Bight	*Mustelus canis*	Smooth dogfish	11	52 − 104	M = 4, F = 6	1
				**62**			

Table S2. Detailed information on PFAS native compounds, surrogates, and internal standards used in this study

(A) Native PFAS compound

#	Analyte	Type	Compound name	Formula	CAS#	Reference SUR	Reference IS	Precursor (m/z)	Product 1 (m/z)	Product 2 (m/z)	MDL* (ng/g dw)
n1	NFDHA	native	Nonafluoro-3,6-dioxaheptanoic acid	C5HF9O4	151772-58-6	$^{13}C_5$-PFHxA	$^{13}C_2$-PFHxA	201	85		0.10
n2	PFBA	native	Perfluorobutanoic acid	C4HF7O2	375-22-4	$^{13}C_4$-PFBA	$^{13}C_3$-PFBA	213	169		0.20
n3	PFMPA	native	Perfluoro-3-methoxypropanoic acid	C4HF7O3	377-73-1	$^{13}C_5$-PFPeA	$^{13}C_2$-PFHxA	229	85		0.10
n4	3:3FTCA	native	3-Perfluoropropyl propanoic acid	C6H5F7O2	356-02-5	$^{13}C_5$-PFPeA	$^{13}C_2$-PFHxA	241	177	117	0.20
n5	PFPeA	native	Perfluoropentanoic acid	C5HF9O2	2706-90-3	$^{13}C_5$-PFPeA	$^{13}C_2$-PFHxA	263	219		0.10
n6	PFMBA	native	Perfluoro-4-methoxybutanoic acid	C5HF9O3	863090-89-5	$^{13}C_5$-PFPeA	$^{13}C_2$-PFHxA	279	85		0.10
n7	HFPO-DA	native	Hexafluoropropylene oxide dimer acid	C6HF11O3	13252-13-6	$^{13}C_3$-HFPO-DA	$^{13}C_2$-PFHxA	285	169	185	0.10
n8	PFBS	native	Perfluorobutanesulfonic acid	C4HF9O3S	375-73-5	$^{13}C_3$-PFBS	$^{18}O_2$-PFHxS	299	80	99	0.05
n9	PFHxA	native	Perfluorohexanoic acid	C6HF11O2	307-24-4	$^{13}C_5$-PFHxA	$^{13}C_2$-PFHxA	313	269	119	0.05
n10	PFEESA	native	Perfluoro(2-ethoxyethane)sulfonic acid	C4HF9O4S	113507-82-7	$^{13}C_5$-PFHxA	$^{13}C_2$-PFHxA	315	135	69	0.10
n11	4:2FTS	native	1H,1H, 2H, 2H-Perfluorohexane sulfonic acid	C6H5F9O3S	757124-72-4	$^{13}C_2$-4:2FTS	$^{18}O_2$-PFHxS	327	307	81	0.20
n12	5:3FTCA	native	2H,2H,3H,3H-Perfluorooctanoic acid	C8H5F11O2	914637-49-3	$^{13}C_5$-PFHxA	$^{13}C_2$-PFHxA	341	237	217	0.20
n13	PFPeS	native	Perfluoropentanesulfonic acid	C5HF11O3S	2706-91-4	$^{13}C_3$-PFHxS	$^{18}O_2$-PFHxS	349	80	99	0.05
n14	PFHpA	native	Perfluoroheptanoic acid	C7HF13O2	375-85-9	$^{13}C_4$-PFHpA	$^{13}C_2$-PFHxA	363	319	169	0.05
n15	ADONA	native	4,8-dioxa-3H-perfluorononanoic acid	C7H2F12O4	919005-14-4	$^{13}C_3$-HFPO-DA	$^{13}C_2$-PFHxA	377	251	85	0.10
n16	PFHxS	native	Perfluorohexanesulfonic acid	C6HF13O3S	355-46-4	$^{13}C_3$-PFHxS	$^{18}O_2$-PFHxS	399	80	99	0.05
n17	PFOA	native	Perfluorooctanoic acid	C8HF15O2	335-67-1	$^{13}C_8$-PFOA	$^{13}C_4$-PFOA	413	369	169	0.20
n18	6:2FTS	native	1H,1H, 2H, 2H-Perfluorooctane sulfonic acid	C8H5F13O3S	27619-97-2	$^{13}C_2$-6:2FTS	$^{18}O_2$-PFHxS	427	407	81	0.20
n19	7:3FTCA	native	3-Perfluoroheptyl propanoic acid	C10H5F15O2	812-70-4	$^{13}C_5$-PFHxA	$^{13}C_2$-PFHxA	441	317	337	0.20
n20	PFHpS	native	Perfluoroheptanesulfonic acid	C7HF15O3S	375-92-8	$^{13}C_8$-PFOS	$^{13}C_4$-PFOS	449	80	99	0.05
n21	PFNA	native	Perfluorononanoic acid	C9HF17O2	375-95-1	$^{13}C_9$-PFNA	$^{13}C_5$-PFNA	463	419	169	0.05
n22	PFOSA	native	Perfluorooctanesulfonamide	C8H2F17NO2S	754-91-6	$^{13}C_8$-PFOSA	$^{13}C_4$-PFOS	498	78	478	0.05
n23	PFOS	native	Perfluorooctanesulfonic acid	C8HF17O3S	1763-23-1	$^{13}C_8$-PFOS	$^{13}C_4$-PFOS	499	80	99	0.05
n24	NMeFOSA	native	N-methyl perfluorooctanesulfonamide	C9H4F17NO2S	31506-32-8	D_3-NMeFOSA	$^{13}C_4$-PFOS	512	219	169	0.05
n25	PFDA	native	Perfluorodecanoic acid	C10HF19O2	335-76-2	$^{13}C_6$-PFDA	$^{13}C_2$-PFDA	513	469	219	0.05
n26	NEtFOSA	native	N-ethyl perfluorooctanesulfonamide	C10H6F17NO2S	4151-50-2	D_5-NEtFOSA	$^{13}C_4$-PFOS	526	219	169	0.05
n27	8:2FTS	native	1H,1H, 2H, 2H-Perfluorodecane sulfonic acid	C10H5F17O3S	39108-34-4	$^{13}C_2$-8:2FTS	$^{18}O_2$-PFHxS	527	507	81	0.20
n28	9Cl-PF3ONS	native	9-chlorohexadecafluoro-3-oxanone-1-sulfonic acid	C8HClF16O4S	756426-58-1	$^{13}C_3$-HFPO-DA	$^{13}C_2$-PFHxA	531	351	83	0.10
n29	PFNS	native	Perfluorononanesulfonic acid	C9HF19O3S	68259-12-1	$^{13}C_8$-PFOS	$^{13}C_4$-PFOS	549	80	99	0.05
n30	PFUnA	native	Perfluoroundecanoic acid	C11HF21O2	2058-94-8	$^{13}C_7$-PFUnA	$^{13}C_2$-PFDA	563	519	219	0.05
n31	NMeFOSAA	native	N-methyl perfluorooctanesulfonamidoacetic acid	C11H6F17NO4S	2355-31-9	D_3-NMeFOSAA	$^{13}C_4$-PFOS	570	419	526	0.05
n32	NEtFOSAA	native	N-ethyl perfluorooctanesulfonamidoacetic acid	C12H8F17NO4S	2991-50-6	D_5-NEtFOSAA	$^{13}C_4$-PFOS	584	419	526	0.05
n33	PFDS	native	Perfluorodecanesulfonic acid	C10HF21O3S	335-77-3	$^{13}C_8$-PFOS	$^{13}C_4$-PFOS	599	80	99	0.05
n34	PFDoA	native	Perfluorododecanoic acid	C12HF23O2	307-55-1	$^{13}C_2$-PFDoA	$^{13}C_2$-PFDA	613	569	269	0.05
n35	NMeFOSE	native	N-methyl perfluorooctanesulfonamidoethanol	C11H8F17NO3S	24448-09-7	D_7-NMeFOSE	$^{13}C_4$-PFOS	616	59		0.50
n36	NEtFOSE	native	N-ethyl perfluorooctanesulfonamidoethanol	C12H10F17NO3S	1691-99-2	D_9-NEtFOSE	$^{13}C_4$-PFOS	630	59		0.50
n37	11Cl-PF3OUdS	native	11-chloroeicosafluoro-3-oxaundecane-1-sulfonic acid	C10HClF20O4S	763051-92-9	$^{13}C_3$-HFPO-DA	$^{13}C_2$-PFHxA	631	451	83	0.10
n38	PFTrDA	native	Perfluorotridecanoic acid	C13HF25O2	72629-94-8	$^{13}C_2$-PFTeDA	$^{13}C_2$-PFDA	663	619	169	0.05
n39	PFDoS	native	Perfluorododecanesulfonic acid	C12HF25O3S	79780-39-5	$^{13}C_8$-PFOS	$^{13}C_4$-PFOS	699	80	99	0.05
n40	PFTA	native	Perfluorotetradecanoic acid	C14HF27O2	376-06-7	$^{13}C_2$-PFTeDA	$^{13}C_2$-PFDA	713	669	169	0.05

(B) PFAS surrogate standards

#	Analyte	Type	Compound name	Abbreviation	Added mass (ng)	Surrogate / Internal standard	Precursor (m/z)	Product 1 (m/z)
s1	MPFBA	isotope	Perfluoro-n-[1,2,3,4-$^{13}C_4$]butanoic acid	$^{13}C_4$-PFBA	4.0	SUR	217	172
s2	M5PFPeA	isotope	Perfluoro-n-[1,2,3,4,5-^{13}C5]pentanoic acid	$^{13}C_5$-PFPeA	2.0	SUR	268	223
s3	M3HFPO-DA	isotope	2,3,3,3-Tetrafluoro-2-(1,1,2,2,3,3,3-heptafluoropropoxy-$^{13}C_3$-propanoic acid	$^{13}C_3$-HFPO-DA	4.0	SUR	287	169
s4	M3PFBS	isotope	Sodium perfluoro-1-[2,3,4-$^{13}C_3$]butanesulfonate	$^{13}C_3$-PFBS	1.0	SUR	302	80
s5	M5PFHxA	isotope	Perfluoro-n-[1,2,3,4,6-^{13}C5]hexanoic acid	$^{13}C_5$-PFHxA	1.0	SUR	318	273
s6	M2-4:2FTS	isotope	Sodium 1H,1H,2H,2H-perfluoro-1-[1,2-$^{13}C_2$]hexane sulfonate	$^{13}C_2$-4:2FTS	2.0	SUR	329	309
s7	M4PFHpA	isotope	Perfluoro-n-[1,2,3,4-$^{13}C_4$]heptanoic acid	$^{13}C_4$-PFHpA	1.0	SUR	367	322
s8	M3PFHxS	isotope	Sodium perfluoro-1-[1,2,3-$^{13}C_3$]hexanesulfonate	$^{13}C_3$-PFHxS	1.0	SUR	402	80
s9	M8PFOA	isotope	Perfluoro-n-[$^{13}C_8$]octanoic acid	$^{13}C_8$-PFOA	1.0	SUR	421	376
s10	M2-6:2FTS	isotope	Sodium 1H,1H,2H,2H-perfluoro-1-[1,2-$^{13}C_2$]-octane sulfonate	$^{13}C_2$-6:2FTS	2.0	SUR	429	409
s11	M9PFNA	isotope	Perfluoro-n-[$^{13}C_9$]nonanoic acid	$^{13}C_9$-PFNA	0.5	SUR	472	427
s12	M8FOSA	isotope	Perfluoro-1-[$^{13}C_8$]octanesulfonamide	$^{13}C_8$-PFOSA	1.0	SUR	506	78
s13	M8PFOS	isotope	Sodium perfluoro-[$^{13}C_8$]octanesulfonate	$^{13}C_8$-PFOS	1.0	SUR	507	80
s14	d3-N-MeFOSA	isotope	N-methyl-d_3-perfluoro-1-octanesulfonamide	D_3-NMeFOSA	2.0	SUR	515	219
s15	M6PFDA	isotope	Perfluoro-n-[1,2,3,4,5,6-$^{13}C_6$]decanoic acid	$^{13}C_6$-PFDA	0.5	SUR	519	474
s16	M2-8:2FTS	isotope	Sodium 1H,1H,2H,2H-perfluoro-1-[1,2-$^{13}C_2$]-decane sulfonate	$^{13}C_2$-8:2FTS	2.0	SUR	529	509
s17	d5-N-EtFOSA	isotope	N-ethyl-d_5-perfluoro-1-octanesulfonamide	D_5-NEtFOSA	1.0	SUR	531	219
s18	M7PFUnA	isotope	Perfluoro-n-[1,2,3,4,5,6,7-$^{13}C_7$]undecanoic acid	$^{13}C_7$-PFUnA	0.5	SUR	570	525
s19	d3-N-MeFOSAA	isotope	N-deuteriomethylperfluoro-1-octanesulfonamidoacetic acid	D_3-NMeFOSAA	2.0	SUR	573	419
s20	d5-N-EtFOSAA	isotope	N-deuterioethylperfluoro-1-octanesulfonamidoacetic acid	D_5-NEtFOSAA	2.0	SUR	589	419
s21	MPFDoA	isotope	Perfluoro-n-[1,2-$^{13}C_2$]dodecanoic acid	$^{13}C_2$-PFDoA	0.5	SUR	615	570
s22	d7-N-MeFOSE	isotope	N-methyl-d_7-perfluorooctanesulfonamidoethanol	D_7-NMeFOSE	10	SUR	623	59
s23	d9-N-EtFOSE	isotope	N-ethyl-d_9-perfluorooctanesulfonamidoethanol	D_9-NEtFOSE	10	SUR	639	59
s24	M2PFTeDA	isotope	Perfluoro-n-[1,2-$^{13}C_2$]tetradecanoic acid	$^{13}C_2$-PFTeDA	0.5	SUR	715	670

(C) PFAS internal standards

#	Analyte	Type	Compound name	Abbreviation	Added mass (ng)	Surrogate / Internal standard	Precursor (m/z)	Product 1 (m/z)
i1	M3PFBA	isotope	Perfluoro-n-[2,3,4-$^{13}C_3$]butanoic acid	$^{13}C_3$-PFBA	1.0	IS	216	172
i2	MPFHxA	isotope	Perfluoro-n-[1,2-$^{13}C_2$]hexanoic acid	$^{13}C_2$-PFHxA	1.0	IS	315	270
i3	MPFHxS	isotope	Perfluoro-1-hexane[$^{18}O_2$]sulfonic acid	$^{18}O_2$-PFHxS	1.0	IS	403	84
i4	MPFOA	isotope	Perfluoro-n-[1,2,3,4-$^{13}C_4$]octanoic acid	$^{13}C_4$-PFOA	1.0	IS	417	172
i5	MPFNA	isotope	Perfluoro-n-[1,2,3,4,5-$^{13}C_5$] nonanoic acid	$^{13}C_5$-PFNA	1.0	IS	468	423
i6	MPFOS	isotope	Sodium perfluoro-1-[1,2,3,4-$^{13}C_4$]octanesulfonate	$^{13}C_4$-PFOS	1.0	IS	503	80
i7	MPFDA	isotope	Perfluoro-n-[1,2-$^{13}C_2$]decanoic acid	$^{13}C_2$-PFDA	1.0	IS	515	470

Table S3. Detailed information on the LC-MS/MS configuration

LC conditions

Parameter	*Value*
LC	Agilent G7120A 1290 Binary Pump<br>Agilent G7116A 1260 Multicolumn Thermostat<br>Agilent G7167A 1260 Multisampler
Analytical column	Agilent ZOBRAX Eclipse Plus C18<br>3.0 x 50 mm, 1.8 µm
Delayed column	Agilent ZOBRAX Eclipse Plus C18<br>4.6 x 50 mm, 3.5 µm
Column temperature	50 °C
Injection volume	5 µL
Mobile phase	A) 5 mM Ammonium acetate in water<br>B) 100% MeOH
Flow rate	0.4 mL/min
Gradient	Time (min) %B<br>0.0 10<br>0.5 10<br>2.0 30<br>14.0 95<br>14.5 100
Stop time	16.5 minutes
Post time	6 minutes

MS conditions

Parameter	*Value*
MS	Agilent 6495 Triple Quadrupole MS/MS<br>Agilent Jet Stream ESI source
Gas Temperature	175 °C
Gas flow	17 L/min
Nebulizer	20 psi
Sheath gas temperature	275 °C
Sheath gas flow	11 L/min
Capillary voltage (Neg)	2500 V
Nozzle voltage (Neg)	0 V
iFunnel<br>High pressure RF (Neg)<br>Low pressure RF (Neg)	 90 V<br>40 V

Table S4. (a) PFAS in the certified reference material IRMM-427 and (b) PFAS surrogate recovery

(a)

	Certified values				Indicative values				
	**PFOS-total**	**PFDA**	**PFUnA**	**PFDoA**	**PFTrDA**	**PFTA**	**PFHxS-total**	**PFNA**	**PFOSA**
	ng/g	*ng/g*	*ng/g*	*ng/g*	*ng/g*	*ng/g*	*ng/g*	*ng/g*	*ng/g*
Certified/indicative value	17 ± 4	1.28 ± 0.17	0.74 ± 0.20	0.97 ± 0.21	0.62 ± 0.29	0.45 ± 0.30	0.09 ± 0.05	0.09 ± 0.05	1.6 ± 0.5
Measured value	20.1 ± 1.5	1.46 ± 0.09	0.81 ± 0.07	1.00 ± 0.13	0.67 ± 0.17	0.53 ± 0.03	1.43 ± 0.17	0.08 ± 0.02	1.63 ± 0.19

Note: 100 mg of CRM tissue used for extraction; each measure value was the average of triplicate (n = 3)

(b)

**Surrogate standards**	**Mean recovery %**	**Standard deviation %**
MPFBA	90%	5%
M5PFPeA	95%	5%
M3PFBS	99%	6%
M2-4-2FTS	90%	26%
M5PFHxA	95%	5%
M3HFPO-DA	80%	5%
M4PFHpA	95%	5%
M3PFHxS	94%	6%
M3-6-2FTS	100%	27%
M8PFOA	97%	6%
M9PFNA	93%	7%
M8PFOS	93%	6%
M2-8-2FTS	84%	22%
M6PFDA	98%	8%
d3-N-MeFOSAA	111%	10%
M7PFUnA	98%	9%
d5-N-EtFOSAA	115%	11%
M8FOSA	77%	9%
M2PFDoA	87%	13%
M2PFTeDA	78%	14%
d3-N-MeFOSA	32%	5%
d7-N-MeFOSE	32%	3%
d9-N-EtFOSE	16%	4%
d-N-EtFOSA	12%	3%

n = 62

Table S5. PFAS concentrations (ng/g ww) in shark muscle tissues reported (a) in this study and (b) in the literature

(a) Present study

Species<br>Common name	Habitat	Stats	PFBA<br>*ng/g*	PFPeA<br>*ng/g*	PFHxA<br>*ng/g*	PFHpA<br>*ng/g*	PFOA<br>*ng/g*	PFOS-L<br>*ng/g*	PFOS*<br>*ng/g*	PFNA<br>*ng/g*	7-3FTCA<br>*ng/g*	PFDA<br>*ng/g*	PFDS<br>*ng/g*	PFUnA<br>*ng/g*	PFOSA<br>*ng/g*	PFDoA<br>*ng/g*	PFTrDA<br>*ng/g*	PFTA<br>*ng/g*	∑ PFAS<br>*ng/g*	Reference
Carcharhinus perezi	The Bahamas	n = 18																		This study
Caribbean reef shark	NW Atlantic Ocean	DF	89%	22%	61%	0%	72%	56%	56%	11%	0%	56%	0%	61%	0%	28%	83%	56%	100%	
		Average	1.10	0.58	0.58		0.98	0.68	0.88	0.19		0.08		0.12		0.10	0.49	0.17	3.33	
		Stdev	0.74	0.48	0.93		1.52	0.67	0.85	0.09		0.05		0.05		0.03	0.22	0.08	2.06	
		Max	3.14	1.00	2.84		5.94	1.99	2.36	0.28		0.17		0.24		0.13	0.91	0.36	9.83	
		Median	0.92	0.59	0.26		0.49	0.38	0.47	0.19		0.06		0.10		0.10	0.45	0.15	3.02	
		Min	0.21	0.12	0.02		0.12	0.04	0.05	0.11		0.03		0.06		0.05	0.23	0.09	1.10	
Alopias vulpinus	New York Bight	n = 8																		This study
Common thresher	NW Atlantic Ocean	DF	100%	63%	100%	13%	100%	75%	75%	0%	88%	100%	100%	100%	0%	100%	100%	100%	100%	
		Average	4.49	1.55	3.38	0.30	6.46	1.78	1.93		1.61	0.54	0.53	3.68		2.44	6.77	1.52	33.7	
		Stdev	3.09	0.87	1.75	0.00	3.61	1.55	1.81		0.98	0.23	0.25	2.04		1.22	5.04	0.96	13.7	
		Max	9.05	2.52	5.82	0.30	10.42	4.84	5.54		3.28	0.81	0.92	5.73		4.04	15.75	3.29	58.5	
		Median	4.48	1.27	3.55	0.30	7.45	1.36	1.41		1.59	0.56	0.44	4.17		2.81	5.87	1.26	31.7	
		Min	1.01	0.56	0.94	0.30	1.90	0.49	0.53		0.46	0.16	0.20	0.64		0.46	0.92	0.34	16.2	
Carcharhinus plumbeus	New York Bight	n = 17																		This study
Sandbar shark	NW Atlantic Ocean	DF	88%	47%	100%	53%	100%	88%	88%	24%	94%	100%	100%	100%	18%	94%	100%	100%	100%	
		Average	1.68	0.38	1.51	0.10	2.45	0.58	0.64	0.04	0.16	0.15	0.24	0.79	0.14	0.69	2.17	0.71	11.1	
		Stdev	0.88	0.21	0.90	0.04	1.46	0.24	0.29	0.01	0.07	0.04	0.09	0.28	0.06	0.24	0.90	0.30	3.58	
		Max	3.17	0.78	3.90	0.19	6.33	1.06	1.27	0.06	0.38	0.27	0.42	1.14	0.19	1.12	3.55	1.11	18.1	
		Median	1.50	0.37	1.37	0.09	1.97	0.66	0.69	0.04	0.14	0.16	0.23	0.84	0.14	0.71	2.31	0.80	11.7	
		Min	0.36	0.10	0.50	0.07	0.76	0.15	0.16	0.03	0.08	0.07	0.07	0.14	0.08	0.29	0.59	0.19	5.38	
Isurus oxyrinchus	New York Bight	n = 8																		This study
Shortfin mako	NW Atlantic Ocean	DF	88%	38%	100%	13%	100%	25%	25%	13%	50%	88%	63%	88%	0%	75%	100%	88%	100%	
		Average	2.04	0.30	2.69	0.18	6.90	0.36	0.40	0.17	0.20	0.16	0.09	0.61		0.49	1.36	0.35	14.5	
		Stdev	1.17	0.20	1.95	0.00	9.15	0.26	0.29	0.00	0.11	0.12	0.05	0.22		0.24	0.60	0.12	10.8	
		Max	3.35	0.49	5.51	0.18	28.6	0.61	0.69	0.17	0.37	0.32	0.17	0.90		0.92	2.77	0.60	38.0	
		Median	2.17	0.30	2.29	0.18	3.99	0.36	0.40	0.17	0.15	0.11	0.07	0.52		0.42	1.21	0.32	12.6	
		Min	0.38	0.10	0.74	0.18	0.95	0.10	0.11	0.17	0.13	0.05	0.05	0.35		0.27	0.93	0.23	4.78	
Mustelus canis	New York Bight	n = 11																		This study
Smooth dogfish	NW Atlantic Ocean	DF	100%	45%	100%	0%	100%	18%	18%	0%	27%	64%	36%	45%	27%	45%	100%	55%	100%	
		Average	2.44	0.70	1.90		4.17	0.11	0.13		0.09	0.12	0.26	0.13	0.08	0.23	0.58	0.32	9.99	
		Stdev	1.52	0.44	1.49		2.93	0.02	0.01		0.02	0.11	0.41	0.03	0.04	0.05	0.23	0.07	5.95	
		Max	6.49	1.20	5.41		10.6	0.14	0.14		0.11	0.37	0.87	0.16	0.13	0.29	0.93	0.37	25.0	
		Median	2.24	0.55	1.57		3.71	0.11	0.13		0.10	0.08	0.06	0.13	0.07	0.23	0.59	0.36	8.62	
		Min	0.90	0.15	0.45		1.12	0.09	0.11		0.07	0.05	0.04	0.09	0.05	0.17	0.31	0.20	4.33	
Total		n = 62																		This study
		DF	92%	40%	89%	18%	92%	56%	56%	11%	48%	79%	55%	77%	10%	65%	95%	77%	100%	
		Average	2.10	0.70	1.85	0.13	3.63	0.78	0.88	0.11	0.50	0.20	0.29	1.02	0.11	0.88	1.96	0.63	12.01	
		Stdev	1.81	0.64	1.59	0.07	4.42	0.86	0.99	0.09	0.77	0.19	0.23	1.48	0.05	0.98	2.75	0.61	11.44	
		Max	9.05	2.52	5.82	0.30	28.6	4.84	5.54	0.28	3.28	0.81	0.92	5.73	0.19	4.04	15.8	3.29	58.51	
		Median	1.59	0.49	1.35	0.10	1.99	0.63	0.69	0.06	0.16	0.14	0.23	0.58	0.10	0.53	0.93	0.37	9.03	
		Min	0.21	0.10	0.02	0.07	0.12	0.04	0.05	0.03	0.07	0.03	0.04	0.06	0.05	0.05	0.23	0.09	1.10	

(b) Previous study

Prionace glauca	SW of Portugal	n = 20													Alves et al., 2016
Blue shark	NE Atlantic Ocean	DF													
		Average	0.023		0.030	0.152		0.007		0.368	0.044			0.624	
		Stdev	0.015		0.129	0.102		0.012		0.191	0.036				
		Max	0.050		0.563	0.461		0.040		0.780	0.120				
		Median													
		Min	<0.010		<0.190	0.046		<0.010		0.130	0.040				
Sphyrna tiburo	Wassaw-Tybee Isla	n = 3													Kumar et al., 2009
Bonnethead shark	NW Atlantic Ocean	DF													
		Average													
		Stdev													
		Max	0.1	0.1	0.4	0.4	0.2		0.2	0.1	0.1				
		Median													
		Min	<0.1	<0.1	<0.1	<0.1	<0.1		<0.1	<0.1	<0.1				
Isurus oxyrinchus	Cretan Sea	n = 1													Zafeiraki et al., 2019
Shortfin mako shark	Mediterranean Sea	DF													
		Average								0.4	0.4	1.9	0.5	3.2	
		Stdev													
		Max													
		Median													
		Min													
Oxynotus centrina	Cretan Sea	n = 1													Zafeiraki et al., 2019
Angular roughshark	Mediterranean Sea	DF													
		Average			0.2	2.8	0.6	0.6		3.6	1.4	7.2	1.6	17.9	
		Stdev													
		Max													
		Median													
		Min													
Mobula mobular	Ionian Sea	n = 1													Zafeiraki et al., 2019
Giant devil ray	Mediterranean Sea	DF													
		Average				0.7				0.1		0.4	0.2	1.4	
		Stdev													
		Max													
		Median													
		Min													
Odontaspis ferox	Aegean Sea (Euboe	n = 2													Zafeiraki et al., 2019
Smalltooth sand tiger	Mediterranean Sea	DF													
		Average			0.1	1.0		0.1		1.2	0.3	3.3	0.5	6.5	
		Stdev			0.1	0.1		0.2		0.1	0.4	0.7	0.2	0.5	
		Max			0.2	1.1		0.2		1.3	0.5	3.8	0.6		
		Median													
		Min			<LOQ	0.9		<LOQ		1.1	<LOQ	2.8	0.3		
Alopias superciliosus	Libyan/Ionian Sea	n = 2													Zafeiraki et al., 2019
Bigeye thresher	Mediterranean Sea	DF													
		Average			0.1	1.1		0.1		2.6	1.3	7.2	1.0	13.4	
		Stdev			0.1	0.5		0.1		2.3	1.2	6.6	0.8	8.0	
		Max			0.2	1.4		0.1		4.2	2.2	11.9	1.6		
		Median													
		Min			<LOQ	0.8		<LOQ		1.0	0.5	2.5	0.5		

Heptranchias perlo	Aegean Sea	n = 4															Zafeiraki et al., 2019
Sharpnose sevengills sha	Mediterranean Sea	DF															
		Average					0.4				0.3			1.1	0.1	1.9	
		Stdev					0.5				0.3			0.8	0.1	1.5	
		Max					0.7				0.6			1.7	0.2		
		Median															
		Min					<LOQ				<LOQ			<LOQ	<LOQ		
Hexanchus griseus	Aegean/Cretan Sea	n = 6															Zafeiraki et al., 2019
Bluntnose sixgills shark	Mediterranean Sea	DF															
		Average			0.1		4.6	0.4	0.5		2.6		0.7	3.7	0.4	12.9	
		Stdev			0.2		8.4	0.6	0.8		4.0		1.3	5.0	0.5	20.8	
		Max			0.6		21.6	1.6	2.2		10.7		3.4	13.8	1.4		
		Median															
		Min			<LOQ		0.3	<LOQ	<LOQ		0.3		<LOQ	0.4	<LOQ		
Prionace glauca	Cretan/Ionian Sea	n = 13															Zafeiraki et al., 2019
Blue shark	Mediterranean Sea	DF															
		Average	0.2				0.1				0.2		0.1	0.8	0.1	1.58	
		Stdev	0.3				0.2				0.2		0.1	0.7	0.1	1.55	
		Max	1.0				0.5				0.9		0.5	2.7	0.5		
		Median															
		Min	<LOQ				<LOQ				<LOQ		<LOQ	0.2	<LOQ		
Carcharhinus leucas	Reunion Island	n = 18															Chynel et al., 2021
Bull shark	SW Indian Ocean	DF															
		Average					0.067	0.0101	0.0162		0.0493		0.0295	0.0803	0.0376	0.2669	
		Stdev					0.142	0.0045	0.0184		0.0255		0.0178	0.0420	0.0281	0.1939	
		Max					0.555	0.0168	0.0751		0.1040		0.0064	0.1578	0.1308	0.8960	
		Median															
		Min					<LOQ	<LOQ	<LOQ		0.0199		<LOQ	0.0268	0.0090	0.0899	
Galeocerdo cuvier	Reunion Island	n = 21															Chynel et al., 2021
Tiger shark	SW Indian Ocean	DF															
		Average					0.0389	0.0070	0.0043		0.0292		0.0133	0.0737	0.0161	0.1443	
		Stdev					0.0614	0.0035	0.0017		0.0093		0.0045	0.0279	0.0066	0.0534	
		Max					0.1480	0.0159	0.0064		0.0463		0.0247	0.1404	0.0320	0.2934	
		Median															
		Min					<LOQ	<LOQ	<LOQ		0.0082		<LOQ	0.0337	<LOQ	0.0690	
Cetorhinus maximus	Ligurian Sea	n = 4															Boldrocchi et al., 2022
Basking shark	Mediterranean Sea	DF															
		Average							0.020		0.22*		0.09	0.34ł	0.06	0.73ł	
		Stdev							0.005		n/a		0.03	n/a	0.03		
		Max															
		Median															
		Min															
Carcharodon carcharias	Nova Scotia/Massac	n = 12															Marciano et al., 2024
White shark	NW Atlantic Ocean	DF															
		Average	0.009	0.034	<0.030	0.031	0.037	<0.024	<0.011	0.004	0.011	0.035	0.024	0.094	0.030	0.388	
		Stdev	0.003	0.026		0.051	0.064			0.004	0.012		0.016	0.092	0.025	0.188	
		Max	0.011	0.100	0.025	0.187	0.234	0.024	0.011	0.014	0.046	0.040	0.055	0.336	0.097	0.839	
		Median	0.007	0.025		0.015	0.017			0.003	0.008		0.028	0.061	0.019	0.319	
		Min	0	0.010		0.004	0.006		0	0.001	0.002	0.030	0.003	0.023	0.012	0.203	

note: DF = detection frequency; PFOS-L = linear PFOS; PFOS* = total PFOS; ł: data extracted from figures

Table S6. Statistical significance between male and female sharks calculated by the Mann-Whitney U test

	PFBA	PFPeA	PFHxA	PFHpA	PFOA	PFOS*	PFNA	7-3 FTCA	PFDA	PFDS	PFUnA	PFOSA	PFDoA	PFTrDA	PFTA	ΣLong-chain PFAS	ΣUltra-long-chain PFAS	ΣPFAS
**Caribbean reef shark**	0.31	n/a	0.88	n/a	0.41	n/a	n/a	n/a	n/a	n/a	n/a	n/a	n/a	0.40	n/a	0.09	0.26	0.32
**Common thresher**	0.61	0.74	0.43	n/a	0.53	0.34	n/a	0.49	0.47	0.68	0.44	n/a	0.22	0.16	0.12	0.37	0.22	0.25
**Sandbar shark**	0.21	0.55	0.45	0.18	0.62	0.15	n/a	0.15	0.89	0.32	0.72	n/a	0.87	0.69	0.42	0.53	0.54	0.17
**Shortfin Mako**	0.88	n/a	0.70	n/a	0.43	n/a	n/a	n/a	0.80	0.58	0.99	n/a	0.71	0.61	0.66	0.48	0.59	0.77
**Smooth dogfish**	0.13	1.00	0.45	n/a	0.32	n/a	n/a	n/a	0.42	n/a	0.59	n/a	0.56	0.83	0.19	0.33	0.94	0.30
**All shark species**	0.47	0.66	0.62	0.20	0.44	0.29	0.48	0.91	0.54	0.63	0.33	n/a	0.26	0.13	0.26	0.14	0.12	0.20

PFOS* = total PFOS (linear + branched)

Table S7. Correlation coefficients examining relationships between length and PFAS concentrations in muscle tissue of five shark species. Bold indicates a statistically significant correlation at α = 0.05

		PFBA	PFPeA	PFHxA	PFHpA	PFOA	PFOS*	PFNA	7-3 FTCA	PFDA	PFDS	PFUnA	PFOSA	PFDoA	PFTrDA	PFTA	ΣLong-chain PFAS	ΣUltra-long-chain PFAS	ΣPFAS
**Caribbean reef shark**	correlation coefficient (r)	-0.363	-0.982	-0.487	n/a	-0.398	0.631	-1.000	n/a	-0.713	n/a	-0.721	n/a	n/a	0.033	-0.060	-0.139	0.304	-0.486
	sample size (n)	9	3	6	0	6	3	2	0	4	0	4	0	1	7	3	7	8	10
	p-value	0.337	0.120	0.328	n/a	0.435	0.565	n/a	n/a	0.287	n/a	0.279	n/a	n/a	0.944	0.962	0.766	0.465	0.155
**Common thresher**	correlation coefficient (r)	0.140	-0.388	-0.280	n/a	-0.453	-0.296	n/a	-0.468	-0.699	-0.558	-0.600	n/a	-0.718	-0.532	-0.588	-0.483	-0.676	-0.600
	sample size (n)	8	5	8	1	8	6	0	7	8	8	8	0	8	8	8	8	8	8
	p-value	0.741	0.518	0.502	n/a	0.260	0.570	n/a	0.289	0.054	0.150	0.116	n/a	**0.045**	0.175	0.126	0.225	0.066	0.116
**Sandbar shark**	correlation coefficient (r)	-0.135	-0.620	-0.156	0.468	-0.128	0.019	0.693	0.219	-0.131	0.490	0.224	-0.789	0.223	0.476	0.454	-0.057	0.425	0.054
	sample size (n)	15	8	17	9	17	15	4	16	17	17	17	3	16	17	17	17	17	17
	p-value	0.632	0.101	0.550	0.204	0.624	0.946	0.307	0.414	0.617	**0.046**	0.386	0.421	0.407	0.054	0.067	0.829	0.089	0.837
**Shortfin mako**	correlation coefficient (r)	0.515	0.971	-0.262	n/a	-0.235	1.000	n/a	-0.086	-0.159	0.051	0.435	n/a	0.013	0.068	0.073	-0.243	0.179	-0.164
	sample size (n)	7	3	8	1	8	2	1	4	7	5	7	0	6	8	7	8	8	8
	p-value	0.237	0.154	0.531	n/a	0.575	n/a	n/a	0.914	0.733	0.936	0.330	n/a	0.980	0.872	0.877	0.562	0.672	0.697
**Smooth dogfish**	correlation coefficient (r)	-0.101	0.857	-0.119	n/a	-0.245	1.000	n/a	1.000	-0.027	-0.829	0.807	1.000	0.827	0.492	-0.435	-0.246	0.385	-0.145
	sample size (n)	10	4	10	0	10	2	0	2	6	3	4	2	4	10	5	10	10	10
	p-value	0.782	0.143	0.743	n/a	0.494	n/a	n/a	n/a	0.960	0.378	0.193	n/a	0.173	0.149	0.464	0.493	0.272	0.689

PFOS* = total PFOS (linear + branched)

Table S8. Correlation coefficients examining relationships between $\delta^{13}C$ and PFAS concentrations in muscle tissue of five shark species. Bold indicates a statistically significant correlation at α = 0.05

		PFBA	PFPeA	PFHxA	PFHpA	PFOA	PFOS-br	PFOS-L	PFOS*	PFNA	7-3 FTCA	PFDA	PFDS	PFUnA	PFOSA	PFDoA	PFTrDA	PFTA	ΣLong-chain PFAS	ΣUltra-long-chain PFAS	ΣPFAS
**Caribbean reef shark**	correlation coefficient (r)	0.344	0.662	-0.031	n/a	-0.232	-0.549	-0.824	-0.773	-1.000	n/a	-0.530	n/a	-0.790	n/a	n/a	-0.904	0.290	-0.664	-0.526	-0.214
	sample size (n)	10	4	7	0	7	4	4	4	2	0	5	0	5	0	1	8	4	8	9	11
	p-value	0.330	0.338	0.947	n/a	0.617	0.451	0.176	0.227	n/a	n/a	0.359	n/a	0.112	n/a	n/a	**0.002**	0.710	0.073	0.146	0.527
**Common thresher**	correlation coefficient (r)	-0.505	-0.587	0.086	n/a	0.265	-0.275	-0.355	-0.366	n/a	-0.123	-0.044	-0.024	0.117	n/a	0.147	0.183	0.047	0.144	0.147	-0.056
	sample size (n)	7	5	7	1	7	5	5	5	0	6	7	7	7	0	7	7	7	7	7	7
	p-value	0.248	0.298	0.855	n/a	0.566	0.654	0.557	0.544	n/a	0.816	0.925	0.959	0.802	n/a	0.753	0.695	0.920	0.757	0.752	0.905
**Sandbar shark**	correlation coefficient (r)	0.092	0.293	-0.022	-0.007	0.008	-0.268	0.440	0.342	-0.214	0.098	-0.029	0.277	0.104	-0.040	0.669	0.294	0.334	0.053	0.210	0.171
	sample size (n)	15	8	16	9	16	14	14	14	4	15	16	16	16	3	15	16	16	16	16	16
	p-value	0.744	0.481	0.935	0.987	0.978	0.354	0.116	0.231	0.786	0.727	0.916	0.300	0.702	0.975	**0.006**	0.269	0.206	0.845	0.435	0.527
**Shortfin mako**	correlation coefficient (r)	-0.612	1.000	0.079	n/a	-0.134	-1.000	-1.000	-1.000	n/a	0.043	-0.115	-0.004	-0.417	n/a	-0.090	0.014	-0.046	-0.119	0.116	-0.212
	sample size (n)	6	2	7	1	7	2	2	2	1	4	6	5	6	0	5	7	6	7	7	7
	p-value	0.196	n/a	0.866	n/a	0.775	n/a	n/a	n/a	n/a	0.957	0.829	0.995	0.411	n/a	0.886	0.976	0.931	0.800	0.804	0.648
**Smooth dogfish**	correlation coefficient (r)	-0.127	-0.866	-0.251	n/a	-0.284	1.000	-1.000	-1.000	n/a	-0.993	-0.368	-0.577	0.563	-0.818	0.447	0.482	0.359	-0.298	0.373	-0.203
	sample size (n)	11	5	11	0	11	2	2	2	0	3	7	4	5	3	5	11	6	11	11	11
	p-value	0.711	0.058	0.456	n/a	0.398	n/a	n/a	n/a	n/a	0.076	0.416	0.423	0.323	0.390	0.451	0.133	0.485	0.374	0.259	0.550
**All NYB sharks (pooled)**	correlation coefficient (r)	-0.054	-0.117	-0.063	-0.542	0.017	-0.281	-0.135	-0.157	-0.444	-0.040	-0.162	-0.101	-0.177	-0.727	-0.031	-0.087	0.034	-0.032	-0.117	-0.095
	sample size (n)	39	20	41	11	41	23	23	23	5	28	36	32	34	6	32	41	35	41	41	41
	p-value	0.742	0.624	0.694	0.085	0.918	0.194	0.538	0.474	0.454	0.838	0.347	0.582	0.316	0.102	0.867	0.587	0.847	0.843	0.468	0.556

PFOS* = total PFOS (linear + branched); PFOS-br = branched PFOS; PFOS-L = linear PFOS

Table S9. Correlation coefficients examining relationships between $\delta^{15}N$ and PFAS concentrations in muscle tissue of five shark species. Bold indicates a statistically significant correlation at $\alpha = 0.05$

		PFBA	PFPeA	PFHxA	PFHpA	PFOA	PFOS-br	PFOS-L	PFOS*	PFNA	7-3 FTCA	PFDA	PFDS	PFUnA	PFOSA	PFDoA	PFTrDA	PFTA	ΣLong-chain PFAS	ΣUltra-long-chain PFAS	ΣPFAS
**Caribbean reef shark**	correlation coefficient (r)	-0.151	-0.342	-0.532	n/a	0.291	0.791	0.965	0.938	1.000	n/a	-0.043	n/a	0.103	n/a	n/a	0.390	-0.598	0.605	0.286	-0.291
	sample size (n)	10	4	7	0	7	4	4	4	2	0	5	0	5	0	1	8	4	8	9	11
	p-value	0.677	0.658	0.219	n/a	0.527	0.209	**0.035**	0.062	n/a	n/a	0.945	n/a	0.869	n/a	n/a	0.340	0.402	0.112	0.456	0.386
**Common thresher**	correlation coefficient (r)	0.146	0.473	-0.008	n/a	-0.127	-0.801	-0.902	-0.913	n/a	0.704	-0.011	0.295	-0.452	n/a	-0.404	-0.570	-0.460	-0.094	-0.457	-0.275
	sample size (n)	7	5	7	1	7	5	5	5	0	6	7	7	7	0	7	7	7	7	7	7
	p-value	0.755	0.421	0.986	n/a	0.786	0.103	**0.036**	**0.030**	n/a	0.119	0.981	0.521	0.308	n/a	0.369	0.181	0.299	0.842	0.303	0.551
**Sandbar shark**	correlation coefficient (r)	0.132	0.066	0.580	-0.265	0.607	0.088	0.684	0.637	0.233	0.306	0.756	0.659	0.721	-0.485	0.699	0.619	0.695	0.692	0.683	0.803
	sample size (n)	15	8	16	9	16	14	14	14	4	15	16	16	16	3	15	16	16	16	16	16
	p-value	0.639	0.876	**0.019**	0.491	**0.013**	0.765	**0.007**	**0.014**	0.767	0.267	**0.001**	**0.005**	**0.002**	0.678	**0.004**	**0.011**	**0.003**	**0.003**	**0.004**	**0.000**
**Shortfin mako**	correlation coefficient (r)	-0.103	1.000	0.354	n/a	0.044	1.000	1.000	1.000	n/a	0.938	0.188	0.643	0.113	n/a	0.612	0.511	0.618	0.061	0.581	0.050
	sample size (n)	6	2	7	1	7	2	2	2	1	4	6	5	6	0	5	7	6	7	7	7
	p-value	0.845	n/a	0.436	n/a	0.925	n/a	n/a	n/a	n/a	0.062	0.722	0.242	0.831	n/a	0.273	0.242	0.191	0.897	0.172	0.915
**Smooth dogfish**	correlation coefficient (r)	-0.406	0.491	-0.428	n/a	-0.369	1.000	-1.000	-1.000	n/a	0.384	-0.289	-0.417	0.139	-0.098	0.219	0.331	0.252	-0.360	0.293	-0.337
	sample size (n)	11	5	11	0	11	2	2	2	0	3	7	4	5	3	5	11	6	11	11	11
	p-value	0.215	0.401	0.189	n/a	0.264	n/a	n/a	n/a	n/a	0.749	0.529	0.583	0.824	0.937	0.723	0.320	0.630	0.276	0.382	0.311
**All NYB sharks (pooled)**	correlation coefficient (r)	-0.060	0.273	-0.073	-0.432	-0.197	0.095	0.198	0.200	-0.865	0.157	0.185	0.393	0.143	0.126	0.193	0.293	0.318	-0.100	0.367	0.076
	sample size (n)	39	20	41	11	41	23	23	23	5	28	36	32	34	6	32	41	35	41	41	41
	p-value	0.715	0.245	0.651	0.184	0.216	0.665	0.365	0.361	0.058	0.425	0.281	**0.026**	0.420	0.812	0.289	0.063	0.062	0.535	**0.018**	0.635

PFOS* = total PFOS (linear + branched); PFOS-br = branched PFOS; PFOS-L = linear PFOS

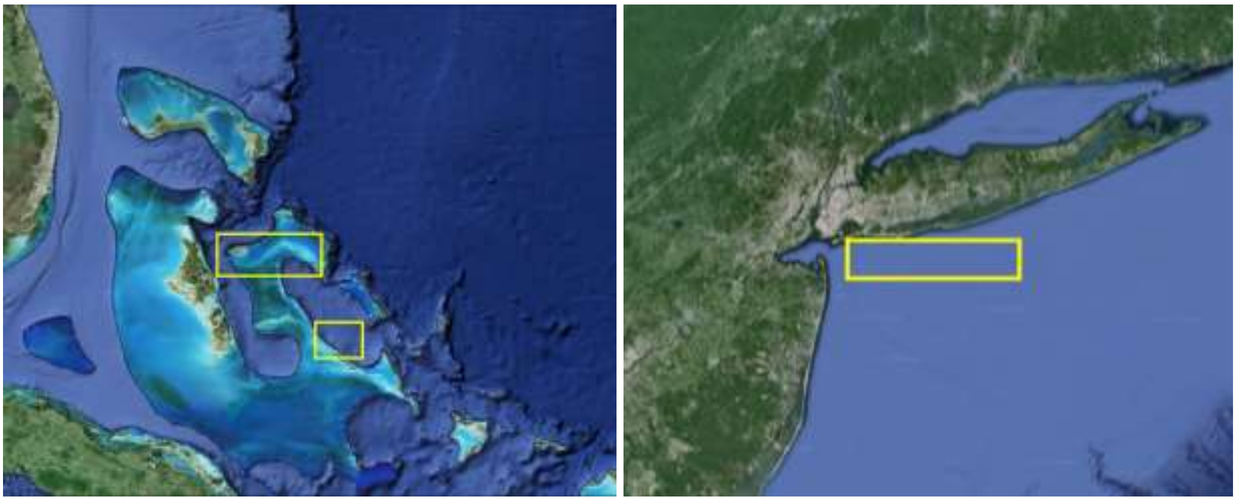

Figure S1. Sampling location (yellow boxes) of sharks from The Bahamas (left panel) and the New York Bight (right panel).

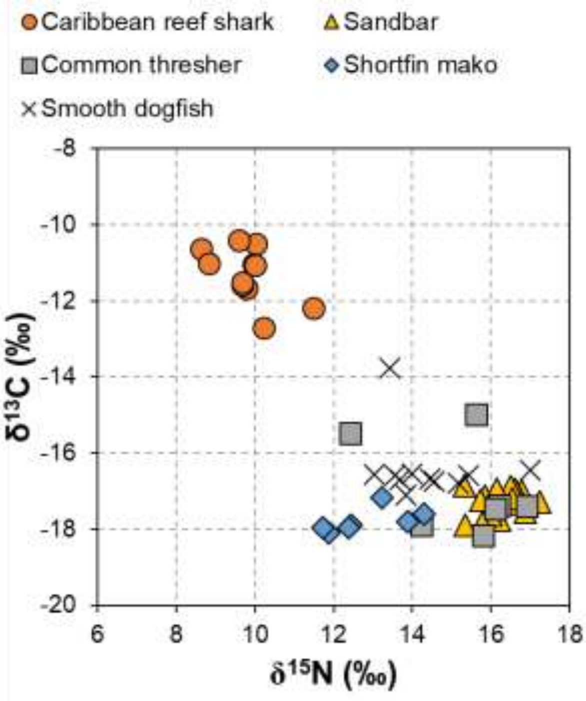

Figure S2. Stable isotope analysis ($\delta^{13}C$ and $\delta^{15}N$) measured in muscle tissue of five shark species.
